# Supplementary material for: Understanding how Victoria, Australia gained control of its second COVID-19 wave
Source: Nat Commun. 2021 Nov 1;12:6266. doi: 10.1038/s41467-021-26558-4 (PMC8560916; doi:10.1038/s41467-021-26558-4)
Supplement: Supplementary file 1 — Supplementary Information [file 41467_2021_26558_MOESM1_ESM.pdf]

Understanding how Victoria, Australia gained control of its second COVID-19 wave, Supplementary Appendix

Contents

1 Base model construction 2

2 Case detection and isolation 7

3 Contact tracing and quarantine 8

4 Implementation of non-pharmaceutical interventions 10

5 Simulation of local NPI implementation during Victoria's second wave 13

6 Between service mixing 15

7 Model initialisation 16

8 Parameters 16

9 Calculation of outputs 25

10 Calibration 26

11 Likelihood function 27

12 Ordinary differential equations 28

13 Sensitivity analyses 29

14 Additional analysis of calibration convergence 31

1 Base model construction

Platform for infectious disease dynamics simulation

We developed a deterministic compartmental model of COVID-19 transmission using the AuTuMN platform, publicly available at https://github.com/monash-emu/AuTuMN/. Our repository allows for the rapid and robust creation and stratification of models of infectious disease epidemiology and includes pluggable modules to simulate heterogeneous population mixing, demographic processes, multiple circulating pathogen strains, repeated stratification and other dynamics relevant to infectious disease transmission. The platform was created to simulate TB dynamics, being an infectious disease whose epidemiology differs markedly by setting, such that considerable flexibility is desirable [1]. We have progressively developed the structures of our platform over recent years, and further adapted it to be sufficiently flexible to permit simulation of other infectious diseases, such as COVID-19. A similar model has been applied to several countries of the Asia-Pacific, with the application to the Philippines (without structure to represent geospatial stratification or contact tracing) previously described.[2]

Base COVID-19 model

Using the base framework of an SEIR model (susceptible, exposed, infectious, removed), we split the exposed and infectious compartments into two sequential compartments each (SEEIIR). The two sequential exposed compartments represent the non-infectious and infectious phases of the incubation period, with the latter representing the "presymptomatic" phase such that infectiousness occurs during three of the six sequential phases. For this reason, "active" is a more accurate term for the two sequential "I" compartments and is preferred henceforward. The two infectious compartments represent early and late phases of active disease, during which symptoms occur if the disease episode is symptomatic, and allow explicit representation of notification, case isolation, hospitalisation and admission to the intensive care unit (ICU). The "active" compartment also includes some persons who remain asymptomatic throughout their disease episode, such that these compartments do not map directly to either persons who are infectious or those who are symptomatic (Figure 1).

The latently infected and infectious presymptomatic periods together comprise the incubation period, with the incubation period and the proportion of this period for which patients are infectious defined by input parameters described below. In general, two sequential compartments can be

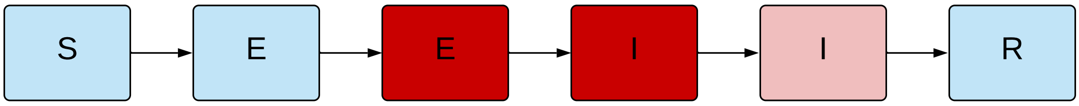

Figure 1: ***Unstratified compartmental model structure.*** *S = susceptible, E = exposed, I = active, R = recovered/removed. Depth of pink/red shading indicates the infectiousness of the compartment.*

used to form a gamma-distributed profile of transition to infectiousness following exposure if the progression rates for these two compartments are equal, although in implementing this model the relative sojourn times in the two sequential compartments usually differed. Nevertheless, the profiles implemented are broadly consistent with the empirically observed log-normal distribution of individual incubation periods [3].

The transition from early active to late active represents the point at which patients are detected (for those persons for whom detection does eventually occur) and isolation then occurs from this point forward (i.e. applies during the late active phase only). This transition point is also used to represent the point of admission to hospital or transition from hospital ward to intensive care for patients for whom this occurs (see Section 1).

Age stratification

All compartments of this base compartmental structure were stratified by age into five-year bands from 0-4 years of age through to 70-74 years of age, with the final age group being those aged 75 years and older. Heterogeneous baseline contact patterns by age were incorporated using age-specific contact rates estimated from survey data from the POLYMOD study, which reported rates of age-specific contacts in various locations between persons of different age groupings (as described below 1). These are then modified by non-pharmaceutical interventions. Our modelled age groups were chosen to match these mixing matrices. The automatic demographic features of AuTuMN that can be used to simulate births, ageing and deaths were not implemented, because the issues considered pertain to the short- to medium-term and the immediate implementation of control strategies, for which population demographics are less relevant.

Contact matrices construction

For each location L (home, school, work, other locations) the age-specific contact matrix $\mathbf{C^L} = (c^L_{i,j}) \in \mathbb{R}^{16\times16}_+$ is defined such that $c^L_{i,j}$ is the average number of contacts that a typical individual aged i has with individuals aged j. As there is no contact survey available for Australia that is complete across all age groups, the matrices $\mathbf{C^L}$ were obtained by extrapolating contact matrices from the United Kingdom, being a country included in the POLYMOD study in 2005 [4]. The original matrices from the United Kingdom are denoted $\mathbf{Q^L} = (q^L_{i,j}) \in \mathbb{R}^{16\times16}_+$, where $q^L_{i,j}$ is defined using the same convention as for $c^L_{i,j}$. The matrices $\mathbf{Q^L}$ were extracted using the R package "socialmixr" (v 0.1.8) and then adjusted to account for age distribution differences between Victoria and the United Kingdom.

Let π_j denote the proportion of people aged j in Victoria, and ρ_j the proportion of people aged j in the United Kingdom. The contact matrices $\mathbf{C^L}$ were obtained from:

$$c^L_{i,j} = q^L_{i,j} \times \frac{\pi_j}{\rho_j}.$$

In sensitivity analysis, we replaced the United Kingdom with Belgium as the proxy country from which survey data were used for constructing Victoria's matrices. Belgium was chosen as the POLYMOD country with the closest similarity in age distribution of Australia and Belgium in 2005.

Clinical stratification

The age-stratified late exposed/incubation and both the early and late active disease compartments were further stratified into five "clinical" categories: *1)* asymptomatic, *2)* symptomatic ambulatory, never detected, *3)* symptomatic ambulatory, ever detected, *4)* ever hospitalised, never critical and *5)* ever critically unwell (Figure 2). The proportion of new infectious persons entering stratum 1 (asymptomatic) is age-dependent. The proportion of symptomatic patients (strata 2 to 5) ever detected (strata 3 to 5) is set through a parameter that represents the time-varying proportion of all symptomatic patients who are ever detected (the case detection rate). Of those ever symptomatic (strata 2 to 5), a time-constant but age-specific proportion is considered to be hospitalised (entering strata 4 or 5). Of those hospitalised (entering strata 4 or 5), a fixed proportion was considered to be critically unwell (entering stratum 5, Figure 3).

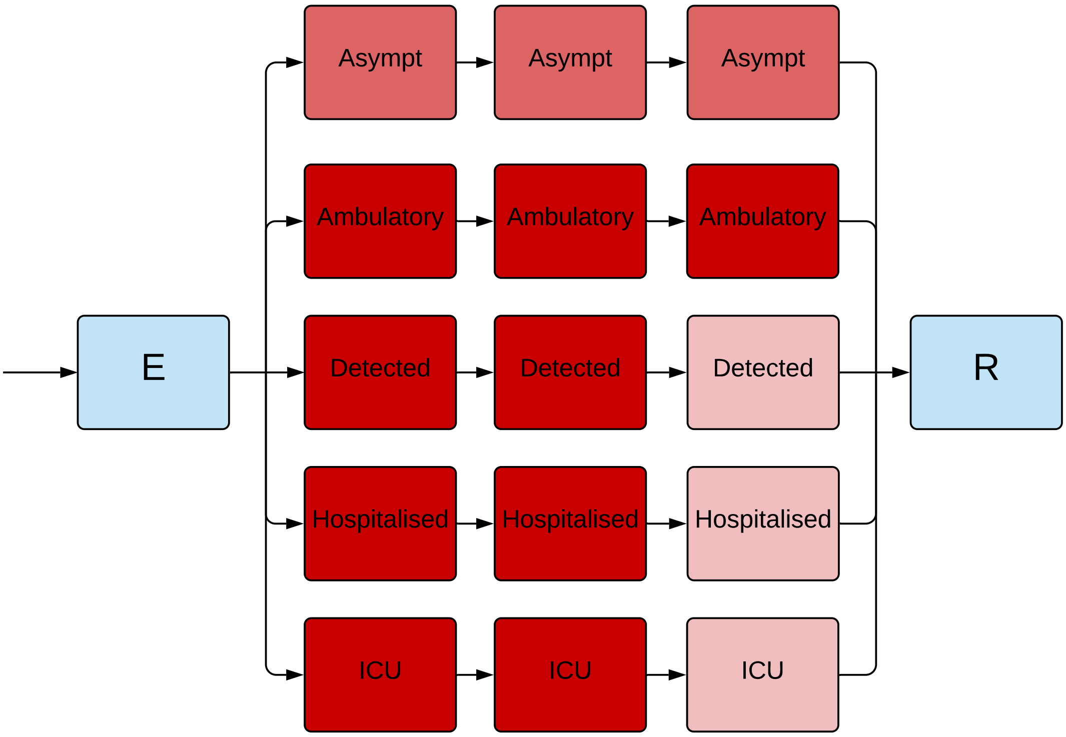

Figure 2: ***Illustration of the implementation of the clinical stratification.*** *Depth of pink/red shading indicates the infectiousness of the compartment. Typical parameter values represented, although the infectiousness of asymptomatic persons is varied in calibration. ICU, intensive care unit.*

Hospitalisation

For COVID-19 patients who are admitted to hospital, the sojourn time in the early and late active compartments is modified, superseding the default values of the sojourn times for these compartments. The point of admission to hospital is considered to be the transition from early to late active disease, such that the sojourn time in the late disease represents the period of time admitted to hospital for patients not admitted to ICU. For patients admitted to ICU, admission to ICU occurs at this same transition point. For this group, the period of time hospitalised prior to ICU admission is estimated as a proportion of the early active period, such that the early active period represents both the period ambulatory in the community and the period in hospital prior

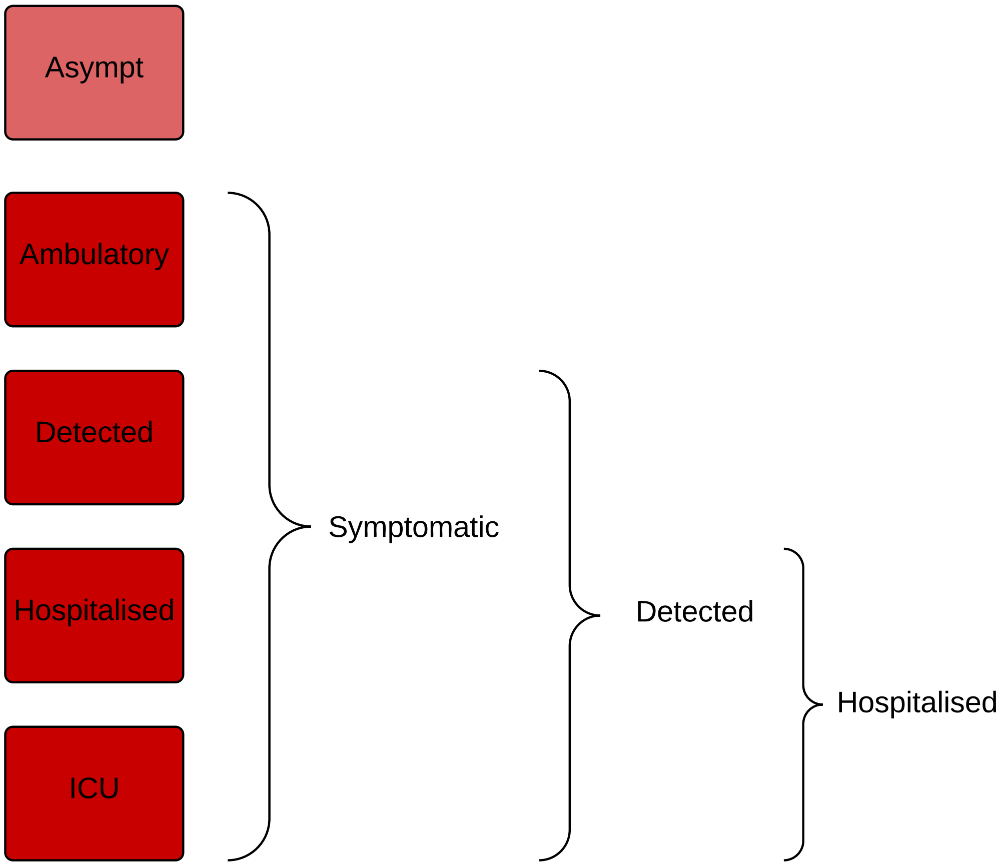

Figure 3: ***Illustration of the rationale for the clinical stratification.***

to ICU admission.

Infectiousness

Asymptomatic persons are assumed to be less infectious per unit time active than symptomatic persons not undergoing case isolation (typically by around 50%, although this is varied in calibration/uncertainty analysis). Infectiousness is also decreased for persons who have been detected to reflect case isolation, and for those admitted to hospital or ICU to reflect infection control procedures (by 80% for both groups). Presymptomatic individuals are assumed to have equivalent infectiousness to those with early active COVID-19.

Application of COVID-19-related death

Age-specific infection fatality rates (IFRs) were applied and distributed across strata 4 and 5, with no deaths typically applied to the first three strata. A ceiling of 50% is set on the proportion of those admitted to ICU (entering stratum 5) who die. If the infection fatality rate is greater than this ceiling, the proportion of critically unwell persons dying was set to 50%, with the remainder of the infection fatality rate then applied to the hospitalised proportion. Otherwise, if the infection fatality rate is less than half of the absolute proportion of persons critically unwell, the infection fatality rate is applied entirely through stratum 5 (such that the proportion of critically unwell persons dying in that age group becomes <50% and the proportion of stratum 4 dying is set to zero). In the event that the infection fatality rate for an age group is greater than the total proportion hospitalised (which is unusual, but could occur for the oldest age group under certain parameter configurations), the remaining deaths are assigned to the asymptomatic stratum. This approach was adopted for computational ease and is valid because the duration active for persons entering this stratum is the same as for the other non-hospitalised strata, such that the dynamics are identical to assigning the deaths to any of the first three strata. We used the age-specific IFRs previously estimated from age-specific death data from 45 countries and results from national-level seroprevalence surveys [5]. Uncertainty in the IFR estimates used in the model is incorporated as described in Section 10.

Clinical stratum	Stratum name	Pre-symptomatic	Early	Late
1	Asymptomatic	0.5	0.5	0.5
2	Symptomatic ambulatory never detected	1	1	1
3	Symptomatic ambulatory ever detected	1	1	0.2
4	Hospitalised never critical	1	1	0.2
5	Ever critically unwell	1	1	0.2

Table 1: ***Illustration of the relative infectiousness of disease compartments by clinical stratification and stage of infection.*** *Typical parameter values displayed.*

2 Case detection and isolation

Determining the proportion of cases detected

We calculate a time-varying case detection rate, being the proportion of all symptomatic cases (clinical strata 2 to 5) that are detected (clinical strata 3 to 5). This proportion is informed by the number of tests performed using the following formula:

$$CDR(time) = 1 - e^{-shape \times tests(time)}$$

time is the time in days from the 31st December 2019 and *tests(time)* is the number of tests per capita done on that date. To determine the value of the shape parameter, we solve this equation based on the assumption that a certain daily testing rate *tests(t)* is associated with a certain *CDR(t)*. Solving for *shape* yields:

$$shape = \frac{-log(1 - CDR(t))}{tests(t)}$$

That is, if it is assumed that a certain daily per capita testing rate is associated with a certain proportion of symptomatic cases detected, we can determine *shape*. As this relationship is not well understood and unlikely to be consistent across all settings, we vary the *CDR* that is associated with a certain per capita testing rate during uncertainty/calibration. Given that the *CDR* value can be varied widely, the purpose of this is to incorporate changes in the case detection rate that reflect the empirical historical profile of changes in testing capacity over time.

The proportion of persons entering clinical stratum 3 is calculated once the *CDR* is known, along with the proportion of all incident cases hospitalised (strata 4 and 5).

Isolation of detected cases

As described in the Section 1 above, as infected persons progress from the early to the late stage of active COVID-19, infectiousness is reduced for those in the detected strata (3 to 5) to reflect case isolation.

3 Contact tracing and quarantine

Model adaptation

We simulate quarantining of persons identified as first degree contacts of COVID-19 patients explicitly through stratification of the compartments representing active COVID-19. That is, the compartments representing both phases of the incubation period and both phases of active COVID-19

are duplicated, with model strata referred to as "traced" and "untraced". In model initialisation, all infectious seed is assigned to the untraced stratum. All newly infected persons commence their incubation period in the untraced stratum of the early incubation period. As for isolated and hospitalised patients, those undergoing quarantine have their infectiousness reduced by 80%.

Contact tracing process

Identification of infected persons through contact tracing is assumed to apply to those in their early incubation period, with flows added to the model that transition persons during their incubation period from the untraced to the traced stratum of this compartment type. The rate of transition from the untraced to the traced stratum of the early incubation period is determined by the proportion of contacts traced. It is assumed that only the contacts of identified cases can be traced, such that the case detection rate (the proportion of symptomatic cases detected) is considered the ceiling for the proportion of contacts traced. The proportion of contacts of identified cases that is traced is multiplied by the proportion of contacts whose index is detected to determine the proportion of all persons entering the incubation period who are traced. The proportion of contacts with detected index, $u(t)$, is calculated as the relative contribution of ever-detected infectious individuals to the total force of infection, given as:

$$u(t) = \frac{\sum_{c \in \mathcal{C}} \sum_{s \in \mathcal{D}} prev_{c,s}(t) \times inf_{c,s}}{\sum_{c \in \mathcal{C}} \sum_{s \in \mathcal{S}} prev_{c,s}(t) \times inf_{c,s}},$$

where $\mathcal{C}$ is the set of infectious compartments, $\mathcal{S}$ represents all clinical strata and $\mathcal{D} \subset \mathcal{S}$ is the list of detected clinical strata. The prevalence of infectious compartment c in clinical stratum s at time t is represented by $prev_{c,s}(t)$, and $inf_{c,s}$ is the relative infectiousness of compartment c in clinical stratum s.

The proportion of contacts of identified cases that is traced, $q(t)$, is considered to decrease as the severity of the COVID-19 epidemic increases, because we expect contact tracing to decline in efficiency as more cases are identified. That is, we assume that contact tracing is universal as COVID-19 prevalence approaches zero and declines exponentially with increasing prevalence. The relationship between the proportion of contacts of identified patients who are quarantined and prevalence is given as:

$$q(t) = e^{-prev(t) \times \tau}$$

Rather than estimate τ directly, we estimate the more intuitive quantity of the proportion of

contacts of identified patients who would be quarantined at a particular prevalence. Solving for the previous equation for τ, we obtain:

$$\tau = \frac{-log(q(t))}{prev(t)}$$

or $\tau = \frac{-log(q_0)}{prev_0}$ at a specific prevalence that accords with a particular value of q. Fixing $prev_0$ at 10^{-3}, we can vary q_0 in calibration as the proportion of contacts of identified cases detected at a prevalence of one active case per thousand population.

Finally, $q(t) \times u(t)$ gives the proportion of all infected persons who are traced. This proportion of persons entering their early latent period transition to the equivalent compartment in the traced stratum before proceeding to the late latent period.

Contact tracing processes

Figure 4 presents the time-varying processes and parameters relating to contact tracing implementation in the model.

Testing data

Statewide daily testing data by date of test were provided by DHHS and applied to all health service clusters to provide a broad profile of the variation in testing capacity over time, including the lower testing numbers in early June compared to at the peak of the epidemic. Data sparseness precluded us from implementing separate functions for each individual health service cluster. For this application to Victoria, the case detection proportion corresponding to a per capita rate of testing of one test per thousand population per day was varied as a calibration parameter in creating the time-varying case detection proportion function. Note that testing rates were typically considerably higher than one per thousand per day during the period modelled, such that the actual modelled case detection proportion is considerably higher than the case detection calibration parameter for most of the simulation period.

4 Implementation of non-pharmaceutical interventions

A major part of the rationale for the development of this model was to capture the past impact of non-pharmaceutical interventions (NPIs) and produce future scenarios projections with the implementation or release of such interventions.

Isolation and quarantine

For persons who are identified with symptomatic disease and enter clinical stratum 3, self-isolation is assumed to occur and their infectiousness is modified as described above. The proportion of ambulatory symptomatic persons effectively identified through the public health response by any means is determined by the case detection rate as described above.

Community quarantine or "lockdown" measures

For all NPIs relating to reduction of human mobility or "lockdown" (i.e. all NPIs other than isolation and quarantine), these interventions are implemented through dynamic adjustments to the age-assortative mixing matrix.

The age-specific mixing matrices we describe above (Section 1) have the major advantage of allowing for disaggregation of total contact rates by location, i.e. home, work, school and other locations. This disaggregation allows for the simulation of various NPIs in the local context by dynamically varying the contribution of each location to reflect the historical implementation of the interventions.

The corresponding mixing matrix (denoted C_0) is presented using the standard convention that a row represents the average number of age-specific contacts per day for a contact recipient of a given age-group. In other words, the element $C_{0i,j}$ is the average number of contacts per day that an individual of age-group $_j$ makes with individuals of age-group $_i$.

This matrix results from the summation of the four location-specific contact matrices: $C_0 = C_H + C_S + C_W + C_L$, where C_H, C_S, C_W and C_L are the age-specific contact matrices associated with households, schools, workplaces and other locations, respectively.

In our model, the contributions of the matrices C_S, C_W and C_L vary with time such that the input contact matrix can be written:

$$C(t) = C_H + s(t)^2 C_S + w(t)^2 C_W + l(t)^2 C_L$$

The modifying functions are each squared to capture the effect of the mobility changes on both the infector and the infectee in any given interaction that could potentially result in transmission. The modifying functions incorporate both macro-distancing and microdistancing effects, depending on the location.

School closures/re-openings

Reduced attendance at schools is represented through the function $s(t)$, which represents the proportion of all school students currently attending on-site teaching. If all education is fully remote, $s(t) = 0$ and C_S does not contribute to the overall mixing matrix $C(t)$. $s(t)$ is calculated through a series of estimates of the proportion of students attending onsite education, to which a smoothed step function is fitted. Note that the dramatic changes in this contribution to the mixing matrix with school closures/re-openings is a more marked change than is seen with the simulation of policy changes in workplaces and other locations (which are determined by empiric data and so do not vary so abruptly or reach a value of zero).

Workplace closures

Workplace closures are represented by quadratically reducing the contribution of workplace contacts to the total mixing matrix over time. This is achieved through the scaling term $w(t)^2$ which modifies the contribution of C_W to the overall mixing matrix $C(t)$. The profile of the function $w(t)$ is set by fitting a polynomial spline function to Google mobility data for workplace attendance (Table 2).

Community-wide movement restriction

Community-wide movement restriction (or "lockdown") measures are represented by proportionally reducing the contribution of the other locations contacts to the total mixing matrix over time. This is achieved through the scaling term $l(t)^2$ which modifies the contribution of C_L to the overall mixing matrix $C(t)$. The profile of the function $l(t)$ is set by fitting a polynomial spline function to an average of Google mobility data for various locations, as indicated in Table 2.

Household contacts

The contribution of household contacts to the overall mixing matrix $C(t)$ is fixed over time. Although Google provides mobility estimates for residential contacts, the nature of these data are different from those for each of the other Google mobility types in that they represent the time spent in that location rather than the number of attendances. The daily frequency with which people attend their residence is likely to be close to one and we considered that household members likely have a daily opportunity for infection with each other household member. Therefore, we did

not implement a function to scale the contribution of household contacts to the mixing matrix with time.

Matrix location	Approach	Google mobility types
School	Policy response	Not applicable
Household	Constant	Not applicable
Workplace	Google mobility	Workplace
Other locations	Google mobility	Unweighted average of:<br>• Retail and recreation<br>• Grocery and pharmacy<br>• Parks<br>• Transit stations

Table 2: ***Mapping of Google mobility data to contact locations .***

Microdistancing

Interventions other than those that prevent people coming into contact with one another are thought to be important to COVID-19 transmission and epidemiology, such as maintaining interpersonal physical distance and the wearing of face coverings. We therefore implemented a "microdistancing" function to represent reductions in the rate of effective contact that is not attributable to persons visiting specific locations and so is not captured through Google mobility data. This microdistancing function reduces the values of all elements of the mixing matrices by a certain proportion. These time-varying functions multiplicatively scale the location-specific contact rate modifiers $s(t)$, $w(t)$ and $l(t)$.

5 Simulation of local NPI implementation during Victoria's second wave

School closures

The effect of Victorian school closures is captured through the timeline presented in Table 3.

Date of change	Policy change	Modification applied to school contacts contribution to mixing matrix, $s(t)$
From model start	Remote learning	0.1
26th May	400,000 school students return to school	0.393
9th June	Remaining 618,000 school students return to school	1
9th July	Remote learning for stage 3 restrictions	0.1

Table 3: ***Timeline used to implement Victorian school closure policies.*** *The function is applied to both metropolitan and regional services.*

Macrodistancing in workplaces and other locations

The functions applied here are determined by the Google mobility data according to Table 2, as described above, but are applied separately for each service. Because Google mobility data pertains to local government areas (LGAs), whereas health service clusters may receive patients from across the state, it was necessary to map mobility data to services. Health service clusters' overall mobility values in each location were calculated using a weighted average of LGA mobility values according to the historical pattern of the origin of patients presenting to services within each service.

As a hypothetical example, if 50% of patients historically presenting to Barwon South West health services come from the City of Geelong, the mobility data for the City of Geelong will contribute 50% of the Google mobility estimate of Barwon South West.

Historical patterns of patient presentations by health service cluster were provided by the Victorian Department of Health and Human Services (DHHS).

Microdistancing approach

In this application to Victoria, the microdistancing function $m(t)$ is comprised of two components: physical distancing and face coverings. Both physical distancing and face coverings microdistancing are applied to the three non-household locations, such that the microdistancing function

for non-household locations is given by:

$$m(t) = d(t)^2 \times f(t)^2$$

The two interventions are assumed to be independent and so are multiplicative. As for the macrodistancing functions, the two functions of time are squared to represent their effects on both the infector and the infectee in any potentially infectious interaction.

Physical distancing

The physical distancing function $d(t)$ is a transposed and translated hyperbolic tan function. The parameters of this function were estimated by using maximum a posteriori inference, with priors that penalised large shape parameters (to avoid extremely rapid transitions). The proportions of respondents answering “always” to YouGov surveys of Victorian residents asking “Thinking about the last 7 days, about how many people from your household have you come into physical contact with (within 2 meters / 6 feet)?” were used as input data. Resulting parameters were: shape, 0.262764; lower asymptote, 0.2803973; upper asymptote; 0.4421819; and inflection point, 15th July. The resulting function is presented in Figure 5.

Face coverings

Two separate face coverings microdistancing functions are employed, one for metropolitan and one for regional health service clusters. These functions were fitted using the same methods as for physical distancing, using YouGov data on Victorian residents’ survey responses to the question “Thinking about the last 7 days, have you worn a face mask outside your home (e.g. when on public transport, going to a supermarket, going to a main road)?”. Estimated parameters were: shape, 0.5261693; lower asymptote, 0.130469; upper asymptote, 0.9143849; and inflection point, 26th July (consistent with the policy change in metropolitan Melbourne). This was applied directly to metropolitan services and translated ten days later for regional services, where face coverings were mandated from the 2nd August. The resulting function is presented in Figure 6.

6 Between service mixing

The preceding section describes the creation of heterogeneous mixing matrices by age for each of the nine health service clusters individually. These mixing matrices are then combined to create a single time-varying heterogeneous mixing matrix by service and age resulting in a 144 by 144

($9 \times 16 = 144$) square mixing matrix. The force of infection for an index service is calculated from the mixing matrices of the age-assortative matrix for each of the services modelled. The spatial mixing matrix is based on the adjacency of health service clusters as indicated in Table 4.

7 Model initialisation

The model was commenced from around one to two weeks earlier than the actual beginning of Victoria's second wave (as determined by genomic analysis), in order that the distribution of infectious persons distributes naturally across compartments as the model approaches the actual beginning of Victoria's second wave in early June. The actual start date selected was the 14th May. The infectious seed needed at this time was then calibrated to ensure dynamics were realistic at the beginning of the second wave. The infectious seed is distributed evenly across metropolitan services, consistent with the epidemic's emergence from Metropolitan Melbourne.

8 Parameters

Non-age-stratified parameters

Parameter	Value	Rationale
Incubation period	Calibration parameter, truncated normal distribution, mean 5.5 days	Estimates of the incubation period have included 5.1 days, 5.2 days and 4.8 days [6, 7, 8, 9]. A systematic review [3] found that data are best fitted by a log-normal distribution (mean 5.8 days, CI 5.0 to 6.7, median 5.1 days). Our systematic review [10] found that estimates of the mean incubation period have varied from 3.6 to 7.4 days.

continuation of parameters table

Proportion of incubation period infectious	50%	Infectiousness is considered to be present throughout a considerable proportion of the incubation period, based on analyses of confirmed source-secondary pairs [11] and early findings that the incubation period was similar to the serial interval [6]. The study of source-secondary pairs was also the primary reference cited by a review of the infectious period that identified studies that quantified the pre-symptomatic period, which concluded that the median pre-symptomatic period could range from less than one to four days [12].
Active period (regardless of detection/isolation, for clinical strata 1 to 3)	Calibration parameter, truncated normal distribution, mean 8 days	This quantity is difficult to estimate, given that identified cases are typically quarantined. Studies in settings of high case ascertainment and an effective public health response have suggested a duration of greater than 5.5 days [9]. PCR positivity, which may continue for up to two to three weeks from the point of symptom onset [11] [12], is difficult to interpret and does not necessarily indicate infectiousness. Consistent with these findings, the duration infectious for asymptomatic persons has been estimated at 6.5 to 9.5 days [12] (although in our model, this would include the pre-symptomatic infectious period).
Proportion of infectious period before isolation or hospitalisation can occur	0.333	Assumed

continuation of parameters table

Disease duration prior to admission for hospitalised patients not critically unwell (i.e. early active sojourn time, stratum 4)	7.7 days	Mean value from ISARIC cohort, as reported on 4th October 2020 in Table 6 [13], and similar to the expected mean from earlier reports from ISARIC [14]. This cohort represents high-income countries better than low and middle-income countries, with the United Kingdom contributing data on the greatest number of patients, followed by France. Earlier estimates of this quantity from China included 4.4 days [6].
Duration of hospitalisation if not critically unwell (late active sojourn time, stratum 4)	12.8 days	Mean value from the ISARIC cohort, as reported on 4th October 2020 in Table 6 [13].
ICU duration (late active sojourn time, stratum 5)	10.5 days	Mean duration of stay in ICU/HDU from ISARIC cohort for patients with complete data, as reported on 10th October 2020 Table 6 [13]. Many other studies reporting on the average duration of ICU stay suffer from right-truncation issues, often estimating 7-10 days length of stay.
Duration of time prior to ICU for patients admitted to ICU	10.5 days	Calculated as the sum of the time from symptom onset to hospital admission (7.7 days above) plus the duration from hospital admission to ICU admission reported by October ISARIC report (2.8 days) [13].
Relative infectiousness of asymptomatic persons (per unit time with active disease)	0.5	Assumed

continuation of parameters table

Relative infectiousness of persons admitted to hospital or ICU	0.2	Assumed
Relative infectiousness of identified persons in isolation	0.2	Assumed
Proportion of hospitalised patients ever admitted to ICU	0.17	Assumed

Table 5: ***Universal (non-age-stratified) model parameters.*** *Point estimates are used as model parameters except where ranges are indicated in calibration parameter table below in calibration table.*

Age-specific parameters

Age-structured parameters are presented in Table 6.

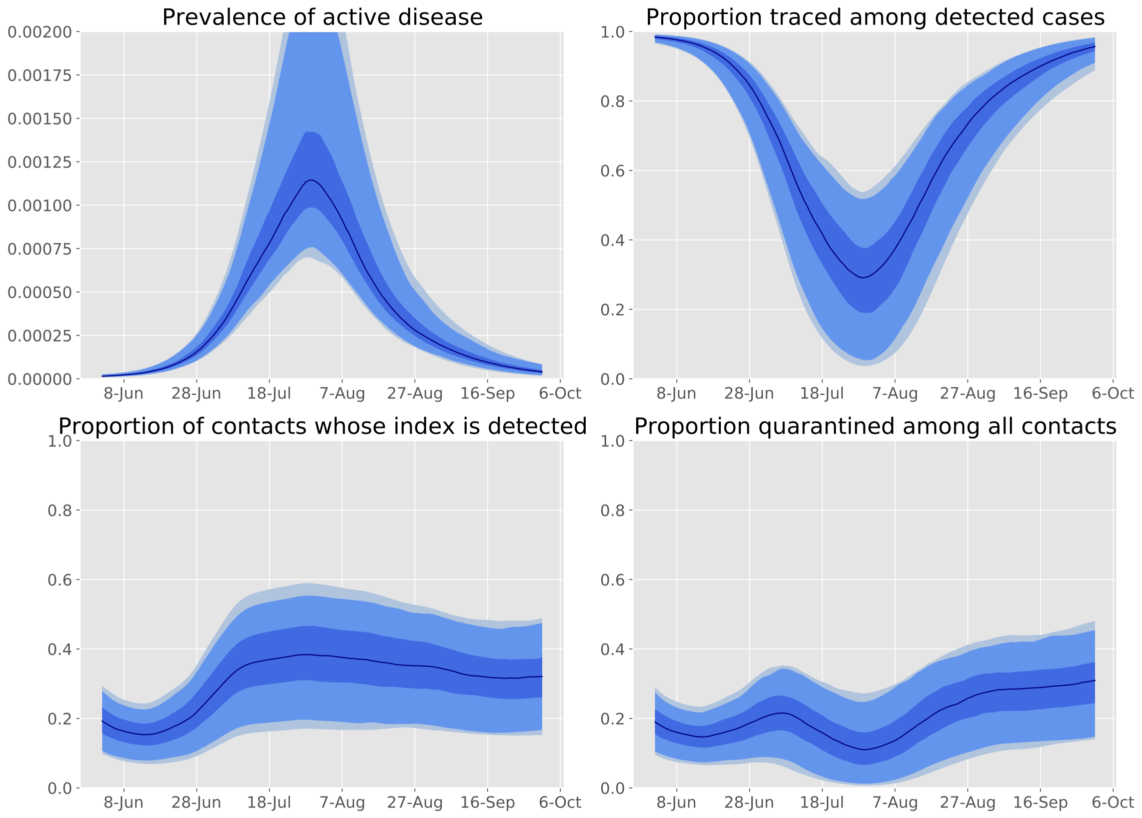

Figure 4: ***Contact tracing time-varying processes and parameters.*** *Upper left panel, modelled prevalence of active COVID-19 episodes (including asymptomatic cases); upper right panel, proportion of contacts traced among contacts of detected cases (declines with increasing prevalence); lower left panel, proportion of all contacts whose index is detected (scales with proportion of symptomatic cases detected, but includes asymptomatic cases); lower right panel, final proportion of all infected persons quarantined through contact tracing. Dark blue line shows the median modelled estimate. Shaded areas represent the 25th to 75th centile (mid blue), 2.5th to 97.5th centile (light blue) and 1st to 99th centile (faintest blue) of estimated detected cases.*

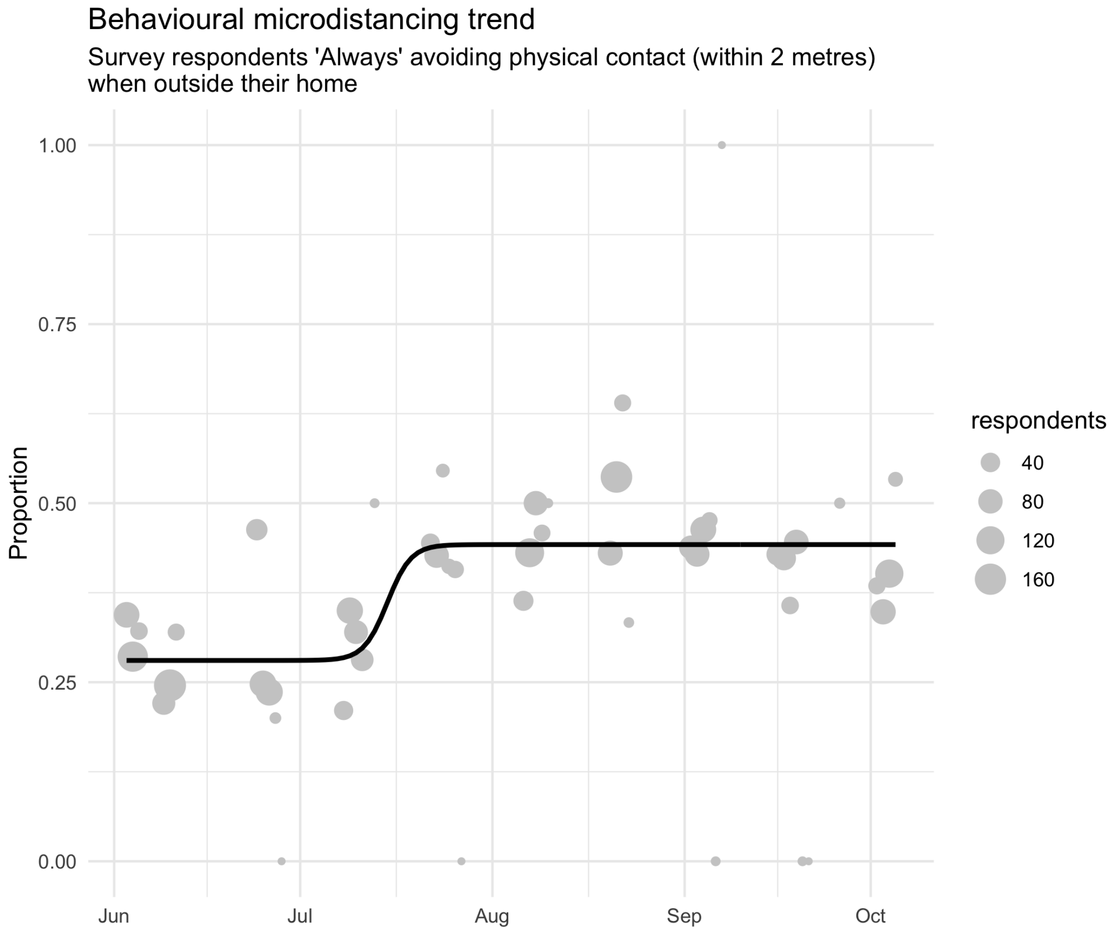

Figure 5: ***Physical distancing micro-distancing function with data used for fitting*** *(for all services).*

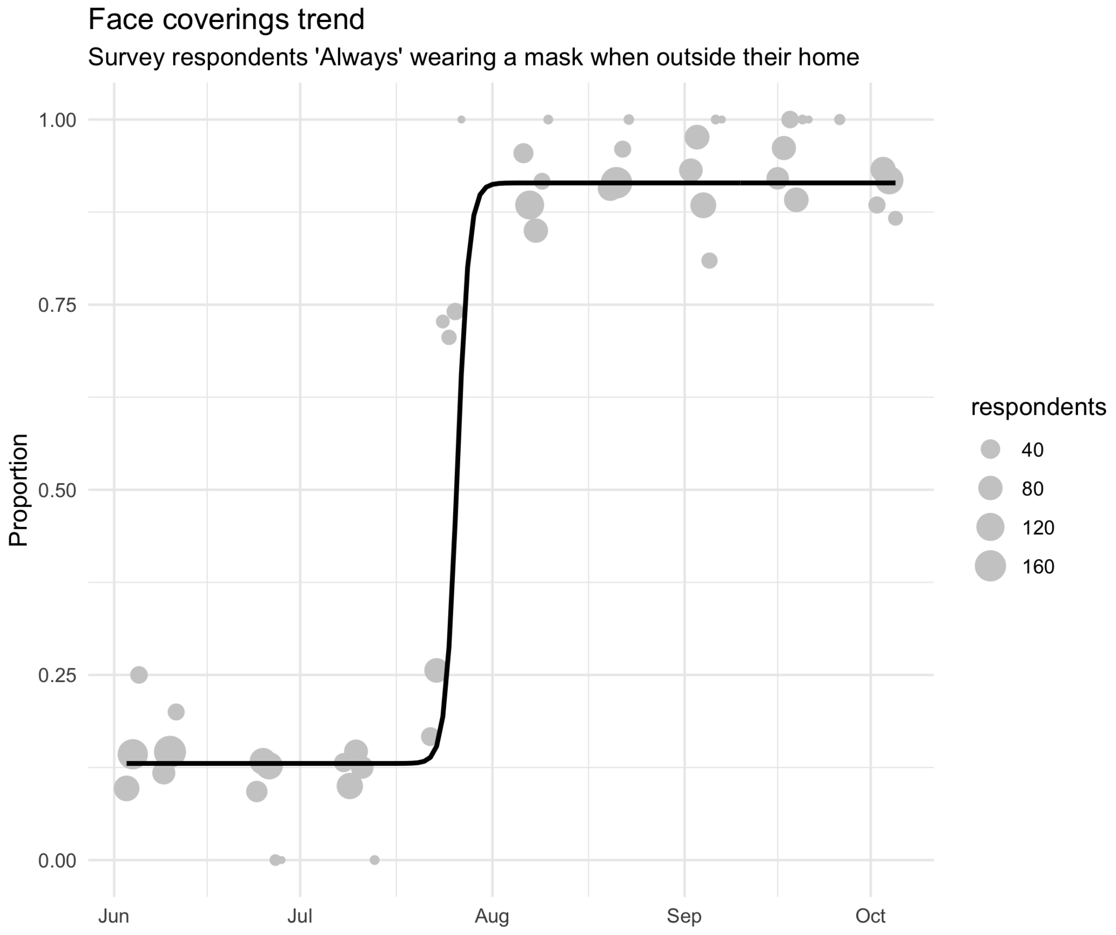

Figure 6: ***Face coverings micro-distancing function for metropolitan Melbourne services with data used for fitting.***

	Barwon South West	Gippsland	Hume	Loddon-Mallee	Grampians	North Metro	South East Metro	South Metro	West Metro
Barwon South West	R	0	0	0	M	M	0	0	M
Gippsland	0	R	M	0	0	0	M	M	0
Hume	0	M	R	M	0	M	M	0	0
Loddon-Mallee	0	0	M	R	M	M	0	0	M
Grampians	M	0	0	M	R	M	0	0	M
North Metro	M	0	M	M	M	R	M	0	M
South East Metro	0	M	M	0	0	M	R	M	0
South Metro	0	M	0	0	0	0	M	R	0
West Metro	M	0	0	M	M	M	0	0	R

Table 4: ***Adjacency-based spatial mixing matrix.*** *0, no mixing between spatial patches; M, calibrated inter-service mixing parameter for adjacent services; R, the diagonal matrix elements are populated with the complement of the other values for each row/column (and so may take a different value in each cell in which it appears)*

Age group (years)	Clinical fraction[a]	Relative susceptibility to infection	Infection fatality rate	Proportion of symptomatic patients hospitalised
0 to 4	0.533	0.36	3×10^{-5}	0.0777
5 to 9	0.533	0.36	$1 \times^{-5}$	0.0069
10 to 14	0.533	0.36	$1 \times^{-5}$	0.0034
15 to 19	0.533	1	$3 \times^{-5}$	0.0051
20 to 24	0.679	1	$6 \times^{-5}$	0.0068
25 to 29	0.679	1	$1.3 \times^{-4}$	0.0080
30 to 34	0.679	1	$2.4 \times^{-4}$	0.0124
35 to 39	0.679	1	$4.0 \times^{-4}$	0.0129
40 to 44	0.679	1	$7.5 \times^{-4}$	0.0190
45 to 49	0.679	1	$1.21 \times^{-3}$	0.0331
50 to 54	0.679	1	$2.07 \times^{-3}$	0.0383
55 to 59	0.679	1	$3.23 \times^{-3}$	0.0579
60 to 64	0.803	1	$4.56 \times^{-3}$	0.0617
65 to 69	0.803	1.41	$1.075 \times^{-2}$	0.1030
70 to 74	0.803	1.41	$1.674 \times^{-2}$	0.1072
75 and above	0.803	1.41	$5.748 \times^{-2}$ [b]	0.0703
Source/ rationale	Table 1 of systematic review and meta-analysis with appropriate accounting for testing during the pre-symptomatic period [15].	Conversion of odds ratios presented in Table S15 of Zhang et al. 2020 to relative risks using data presented in Table S14 of the same study [6].[c]	Estimated from pooled analysis of data from 45 countries from Table S3 of O'Driscoll et al [5]. Values consistent with previous estimates using serosurveys performed in Spain [16].	Estimates from the Netherlands as the first wave of infections declined from 4th May to 21st July [17].

Table 6: ***Age-stratified parameters not varied during calibration, or varied through a common multiplier.*** *[a]Proportion of incident cases developing symptoms. [b]Weighted average of IFR estimates for 70 to 79 and 80 and above age groups. [c]Note the relative magnitude of these values are similar to those estimated by the analysis we use to estimate the age-specific clinical fraction.*

9 Calculation of outputs

Incidence

Incidence is calculated as any transitions into the early active compartment (*"I"*).

Hospital occupancy

This is calculated as the sum of three quantities:

1. All persons in the late active compartment in clinical stratum 4, representing those admitted to hospital but never critically unwell.

2. All persons in the late active compartment in clinical stratum 5, representing those currently admitted to ICU.

3. A proportion of the early active compartment in clinical stratum 5, representing those who will be admitted to ICU at a time in the future. This proportion is calculated as the quotient of *1)* the difference between the pre-ICU period and the pre-hospital period for clinical stratum 4, divided by *2)* the total pre-ICU period. That is, a proportion of the pre-ICU period is considered to represent patients in hospital who have not yet been admitted to ICU.

ICU occupancy

This is calculated as all persons in the late active compartment in clinical stratum 5.

Seropositive proportion

This is calculated as the proportion of the population in the recovered (*"R"*) compartment. Although very similar numerically to the attack rate, persons who died of COVID-19 are not included in the denominator.

COVID-19-related mortality

This is calculated as all transitions representing death, exiting the model. This is implemented as depletion of the late active compartment.

Notifications

Local case notifications are calculated as transitions from the early to the late active compartment for clinical strata 3 to 5.

10 Calibration

We calibrated the model using the adaptive Metropolis algorithm described by Haario et al. [18]. A standard Metropolis algorithm with fixed proposal distribution parameters was used for the first 500 iterations to initiate the covariance matrix before the adaptive algorithm commenced. Seven chains were then run to ensure 10,000 iterations post-burn-in were achieved.

Rationale for service-specific targets

For all services (both metropolitan and regional), we included the time series of daily notifications for that service as a calibration target, using a normal distribution for the likelihood function. A normal distribution is preferred because the mapping process for the notifications for each service results in these quantities not being integer-valued.

In addition, we include time series for the following quantities at the state level. Because these quantities are counts, Poisson distributions are used in likelihood calculations:

- Daily new COVID-19 notifications
- Daily new hospital admissions
- Daily new ICU admissions
- Daily deaths

Assigning targets to services

Hospital admissions and ICU admissions can be mapped directly to a health service cluster. Health service clusters include all health care (including public hospitals, private, rehab, acute, mental health, etc.) and some metropolitan services have changed service assignment over the years. Mapping was performed as at August 2020. However, for the other two indicators used (notifications and deaths), mapping was not possible because these events do not necessarily occur within a health service cluster. Therefore, the local government area (LGA) of residence of the person notified or dying is considered. Each notification and death is split proportionately across the health

service clusters to which they would typically present, according to historical data on hospital presentations for each LGA provided by DHHS. (Note that only notifications are considered as calibration targets, although these considerations are relevant to the comparison between data and modelled outputs undertaken for validation purposes.)

Variation of age-specific proportion hospitalised using "adjuster" parameters

Our parameters included the age-specific proportions of cases hospitalised, which were varied up and down together during calibration. These proportion parameters were modified through an "adjuster" parameter that is not strictly a multiplier, but is rather implemented in such a way as to scale the base parameter value while ensuring that the adjusted parameter remains a proportion (with range zero to one). This adjuster parameter can be considered as a multiplicative factor that is applied to the odds ratio that is equivalent to the baseline proportion to be adjusted. Specifically, the adjusted proportion is equal to:

$$\frac{proportion \times adjuster}{proportion \times (adjuster - 1) + 1}$$

Variation of infection fatality rate

The infection fatality rate can be defined as the risk of death given an episode of infection, including asymptomatic and undetected episodes. This is considered a more stable quantity than the case fatality rate. However, it is still likely to vary considerably between settings and so is adjusted during the calibration process. Because the epidemic in Victoria was characterised by high rates of transmission and disease in aged care, we fix the infection fatality rate for all age groups up to 74 years to the estimates derived from the literature, but vary the infection fatality rate for those aged 75 and above. This is intended to capture the increased risk of death for those in residential aged care during the second wave, the large majority of whom would be included in this age bracket.

11 Likelihood function

Likelihood functions are derived from comparing model outputs to target data at each time point nominated for calibration.

The composite likelihood function is given formally as:

$$\prod_{t} n_t(\theta) d_t(\theta) h_t(\theta) i_t(\theta) \times \prod_{t,g} n_{t,g}(\theta, \sigma)$$

where $_t$ indexes the date, $_g$ indexes the service, n_t refers to daily new notifications, d_t to daily deaths, h_t to daily new hospitalisations and i_t to daily new ICU admissions. The contributions of each state-wide component to the composite likelihood are measured with Poisson distributions (e.g. $n_t(\theta) = Poiss(\nu_t(\theta))$, where $\nu_t(\theta)$ is the number of notifications simulated by the model at date t under parameter set θ), and normal distributions are used for each $n_{t,g}$ (because these targets are not integer-valued). σ is the ratio of the peak of each service-specific notification to the corresponding standard deviation of each of the normal distributions used in calculating their contribution to the likelihood. This was included as a calibration parameter to improve calibration efficiency.

12 Ordinary differential equations

For the clearest description of the model, we refer the reader to our code repository, because our object-oriented approach to software development is intended to be highly transparent and readable. For those who prefer dynamical systems such as this presented in the form of ordinary differential equations, we present the following.

$$\frac{dS_{a,g}}{dt} = -\lambda_{a,g}(t)\sigma_a S_{a,g}$$

$$\frac{dE_{a,g,q=1}}{dt} = \lambda_{a,g}(t)\sigma_a S_{a,g} - \alpha E_{a,g,q=1} - \chi(t)E_{a,g,q=1}$$

$$\frac{dE_{a,g,q=2}}{dt} = -\alpha E_{a,g,q=2} + \chi(t)E_{a,g,q=1}$$

$$\frac{dP_{a,c,g,q}}{dt} = p_{a,c}(t)\alpha E_{a,g,q} - \nu P_{a,c,g,q}$$

$$\frac{dI_{a,c,g,q}}{dt} = \nu P_{a,c,g,q} - \gamma_c I_{a,c,g,q}$$

$$\frac{dL_{a,c,g,q}}{dt} = \gamma_c I_{a,c,g,q} - \delta_{a,c} L_{a,c,g,q} - \mu_{a,c} L_{a,c,g,q}$$

$$\frac{dR_{a,g}}{dt} = \sum_{c,q} \delta_{a,c} L_{a,c,g,q}$$

where

$$\lambda_{a,g} = \beta \sum_{g'} \mathbf{G}_{g,g'} \sum_{j,c} \frac{\epsilon P_{j,c,g'}(t) + \iota_c I_{j,c,g'}(t) + \kappa_c L_{j,c,g'}(t)}{N_{j,g'}(t)} C_{a,j}(t)$$

$$\sum_c p_{a,c}(t) = 1, \forall t \in \mathbb{R}$$

$$\chi(t) = \frac{\alpha q(t) u(t)}{1 - q(t) u(t)}$$

$$\mathbf{C}_0 = \mathbf{C}_H + \mathbf{C}_S + \mathbf{C}_W + \mathbf{C}_L$$

$$\mathbf{C}_g(t) = \mathbf{C}_H + s_g(t)^2\mathbf{C}_S + w_g(t)^2\mathbf{C}_W + l_g(t)^2\mathbf{C}_L$$

$$l_g(t) = \frac{re_g(t) + gr_g(t) + pa_g(t) + tr_g(t)}{4}$$

Symbol	Explanation
S	Persons susceptible to infection
E	Persons in the non-infectious incubation period
P	Persons in the incubation period
I	Persons in the early active disease period, before isolation or hospitalisation may occur
L	Persons in the late active disease period, after isolation or hospitalisation may have occurred
R	Persons in the recovered period, from which re-infection cannot occur

13 Sensitivity analyses

Sensitivity analysis using alternative base mixing matrices

We ran a sensitivity analysis using age-specific mixing matrices derived from survey data for Belgium, rather than the United Kingdom, which were weighted to the Victorian age structure using the same methods as described above. The results of this analysis were similar to that of the baseline analysis and are presented in Figure 21, Figure 22 and Figure 23. These results show no significant epidemiological differences from those derived from the base case analysis.

Sensitivity analysis with Google residential mobility used to scale home location contribution to mixing matrices

We ran an alternative analysis, in which the home location contribution to the dynamic mixing matrices scaled with Google residential mobility data. This replaced the baseline assumption that

Symbol	Explanation
t	Time
a	Compartment of age group a
c	Compartment of clinical stratification c
g	Compartment of geographical service stratification g
q	Compartment of tracing stratification q
α	Rate of progression from non-infectious to infectious incubation period
ν	Rate of progression from infectious incubation to early active disease
γ	Rate of progression from early active disease to late active disease
μ	Rate of disease-related death
ϵ	Relative infectiousness of pre-symptomatic compartment
ι	Clinical stratification infectiousness vector for early active compartment
κ	Clinical stratification infectiousness vector for late active compartments
β	Probability of infection per contact between an infectious and susceptible individual
j	Infectious populations
p	Proportion progressing to each clinical stratification
**G**	Square matrix of dimensions 9×9 for nine services, as presented in Table 4

the rates of home location contacts remained fixed throughout the simulations. The results of this analysis were similar to that of the baseline analysis and are presented in Figure 24, Figure 25 and Figure 26.

With this approach, the posterior distributions for the incubation period and the duration active shortened well into the lower tail of their respective normal prior distributions. Meanwhile, the effect of face coverings was clustered towards the upper bound of its prior distribution. Exploring the model through manual calibration, the reason for this was determined to be that it was not possible to achieve a sufficiently steep decline in case rates without shortening the serial interval and emphasising the effect of face coverings. This remained the case even if the effect of face coverings was allowed to reach extremely high (and implausible) levels (e.g. 100%). This is because the importance of home contacts increased towards the peak of the epidemic, as Google residential mobility increased. We do not consider this realistic because these contacts would likely have

Symbol	Explanation
**C**	Mixing matrix
**H**	Household contribution to mixing matrix
**W**	Workplace contribution to mixing matrix
**O**	Other locations contribution to mixing matrix
**S**	Schools contribution to mixing matrix
l	Other locations macrodistancing function of time
w	Function fit to Google mobility data for workplaces
s	Function fit to Google mobility data for schools
re	Function fit to Google mobility data for retail and recreation
gr	Function fit to Google mobility data for grocery and pharmacy
pa	Function fit to Google mobility data for parks
tr	Function fit to Google mobility data for transit stations

been saturated for those in households with active cases, given that Google residential mobility represents the time spent at home, rather than the number of visits taken to a household. Further, we consider the posterior distributions for the incubation period and duration active too short to be realistic under this model configuration.

14 Additional analysis of calibration convergence

In the main analysis described above, each of the seven chains of the adaptive Metropolis algorithm was initialised by adding random noise around parameter values obtained from a previous calibration. This approach was used to assist the algorithm to reach the parameter space's high posterior areas within a reasonable number of iterations. The high number of dimensions ($N = 18$) made other approaches such as Latin-Hypercube-based initialisation impractical, as chains could take millions of iterations before adequately sampling plausible parameter sets.

In a separate experiment, we ran another adaptive Metropolis algorithm where only ten parameters were varied, but this time initialisation was performed with Latin Hypercube Sampling (LHS) across ten independent chains. The ten parameters were selected based on their expected influence on the main findings and whether they were among the primary estimands of interest (e.g. the face coverings effect parameter). The remaining eight parameters were fixed to the maximum a-posteriori value obtained in the main analysis. The aim of this analysis was to verify that

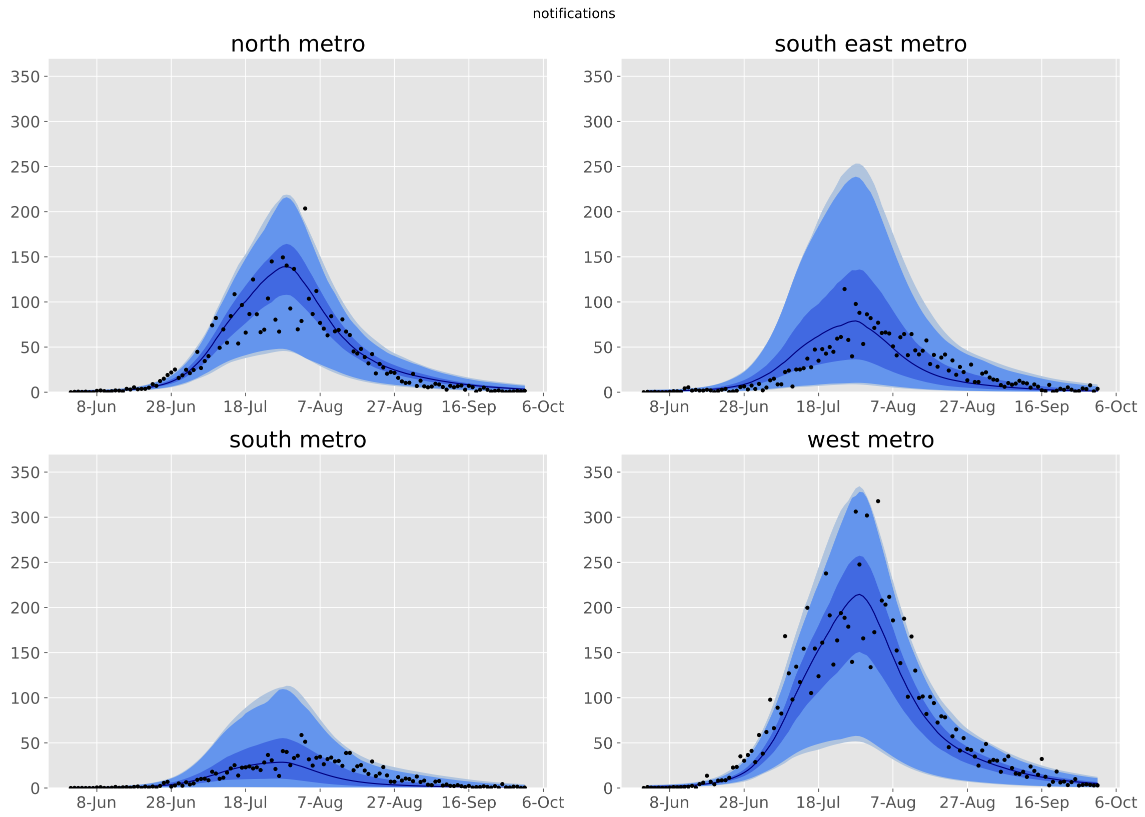

Figure 7: ***Calibration fit to daily time series of notifications for each metropolitan health service cluster.*** *Daily confirmed cases (black dots) overlaid on the median modelled detected cases (dark blue line), with shaded areas representing the 25th to 75th centile (mid blue), 2.5th to 97.5th centile (light blue) and 1st to 99th centile (faintest blue) of estimated detected cases.*

the calibration algorithm could recover similar convergence to that obtained in the main analysis despite starting from highly diverse initial points.

Figures 27 and 28 illustrate the progression of the parameters during this experiment. The parameter traces were compared against the posterior distributions obtained from the main analysis (Figure 28). We found that the chains converged to similar values when considering the LHS-based calibration compared to the original analysis. The posterior ranges obtained with the LHS-based analysis were sometimes slightly narrower compared to the original calibration. This is attributable to the fact that more parameters were fixed throughout calibration in the LHS-based calibration than in the original calibration. Consequently, more constraints were imposed on the varied parameters that were previously found to be correlated with parameters that have been

**Parameter**	**Convergence statistic** ($\hat{R}$)
incubation period	1.022
duration active	1.03
north and west metro	1.036
south and south east metro	1.012
barwon south west	1.04
regional	1.033
infection risk per contact	1.071
inter-service mixing	1.006
infectious seed	1.013
asympt infect multiplier	1.036
75 and above IFR	1.005
hospitalisation adjuster	1.007
CDR at base testing rate	1.016
pre-ICU period	1.016
physical distancing	1.036
face coverings	1.005
home contacts reduction	1.004
target output ratio	1.006
traced prop high prevalence	1.009

Table 7: ***Chain convergence statistics.***

fixed in the LHS-based analysis.

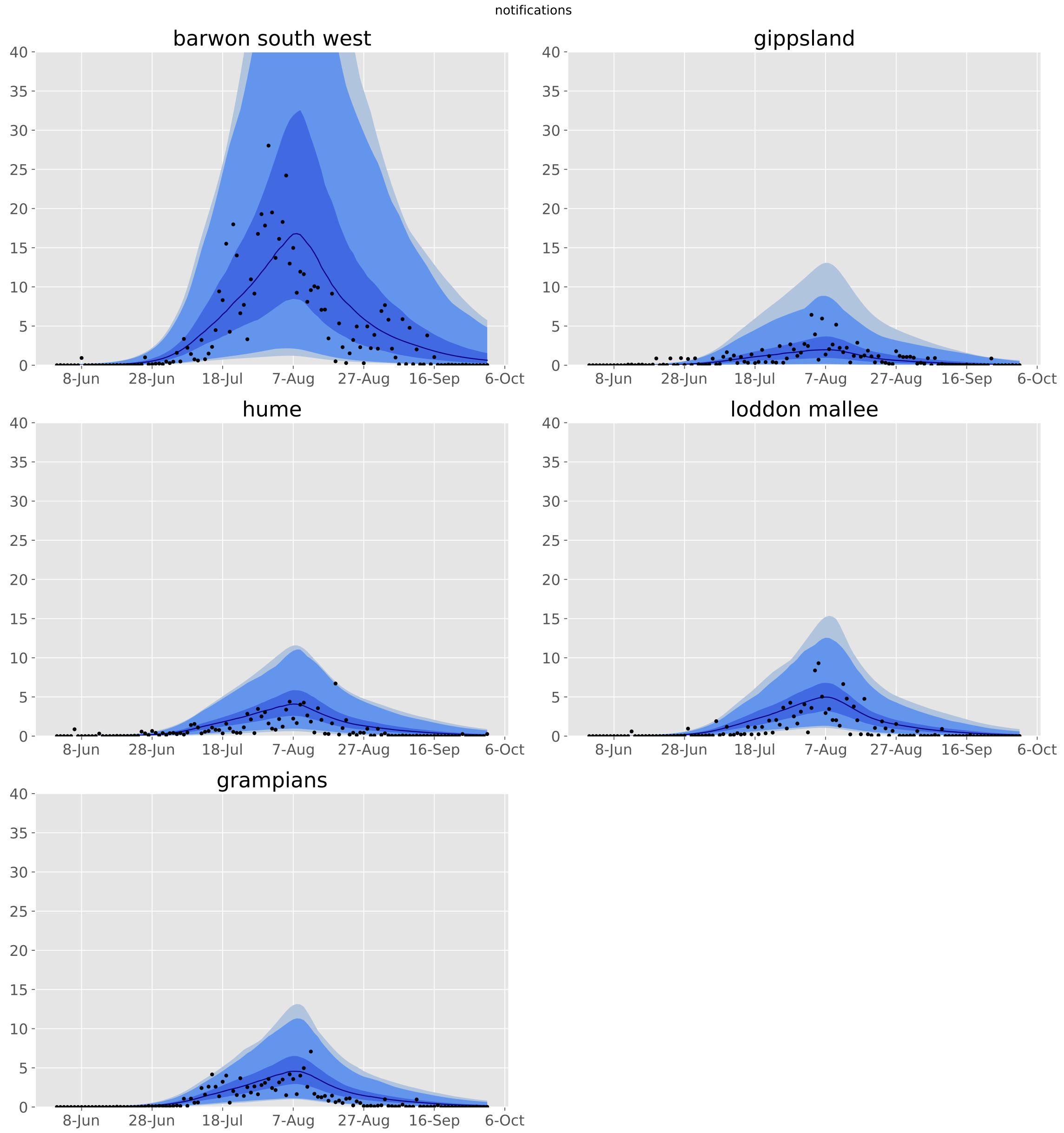

Figure 8: ***Calibration fit to daily time series of notifications for each regional health service cluster.*** *Daily confirmed cases (black dots) overlaid on the median modelled detected cases (dark blue line), with shaded areas representing the 25th to 75th centile (mid blue), 2.5th to 97.5th centile (light blue) and 1st to 99th centile (faintest blue) of estimated detected cases.*

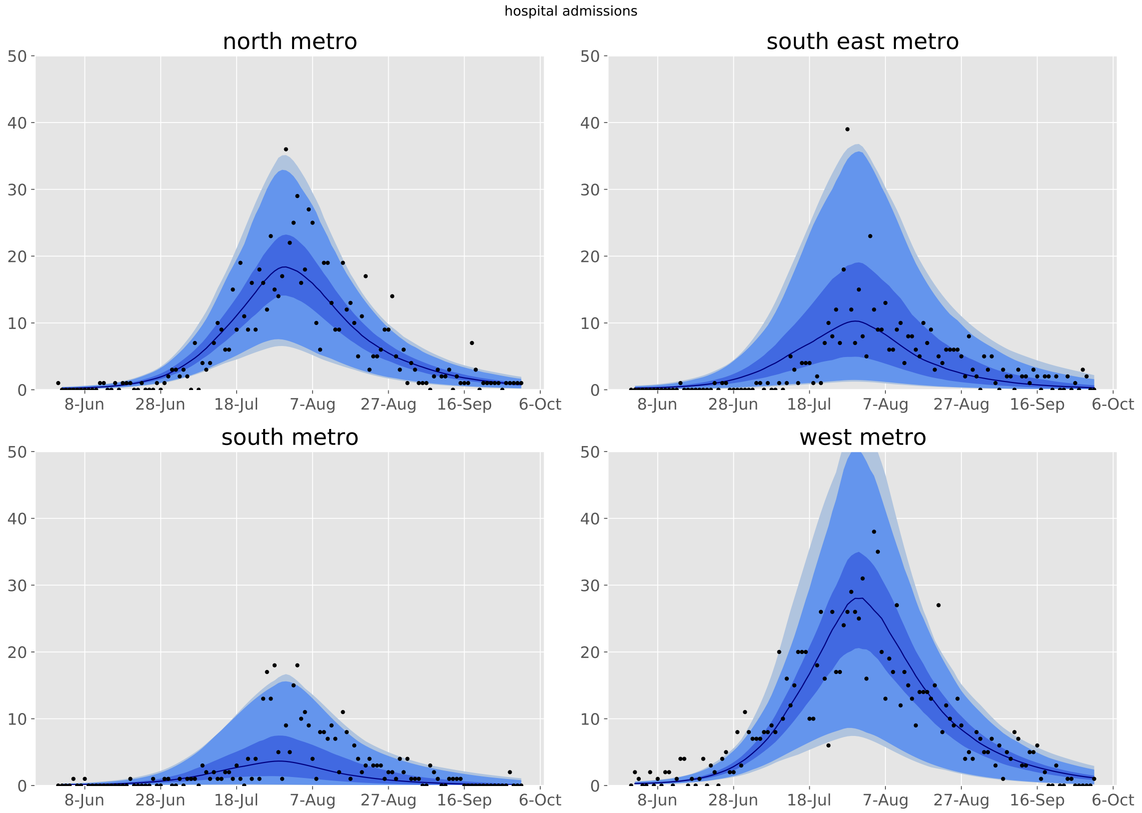

Figure 9: ***Validation fit to daily time series of hospitalisations for each metropolitan health service cluster.*** *Daily hospitalisation (black dots) overlaid on the median modelled hospitalisations (dark blue line), with shaded areas representing the 25th to 75th centile (mid blue), 2.5th to 97.5th centile (light blue) and 1st to 99th centile (faintest blue) of estimated hospitalisations.*

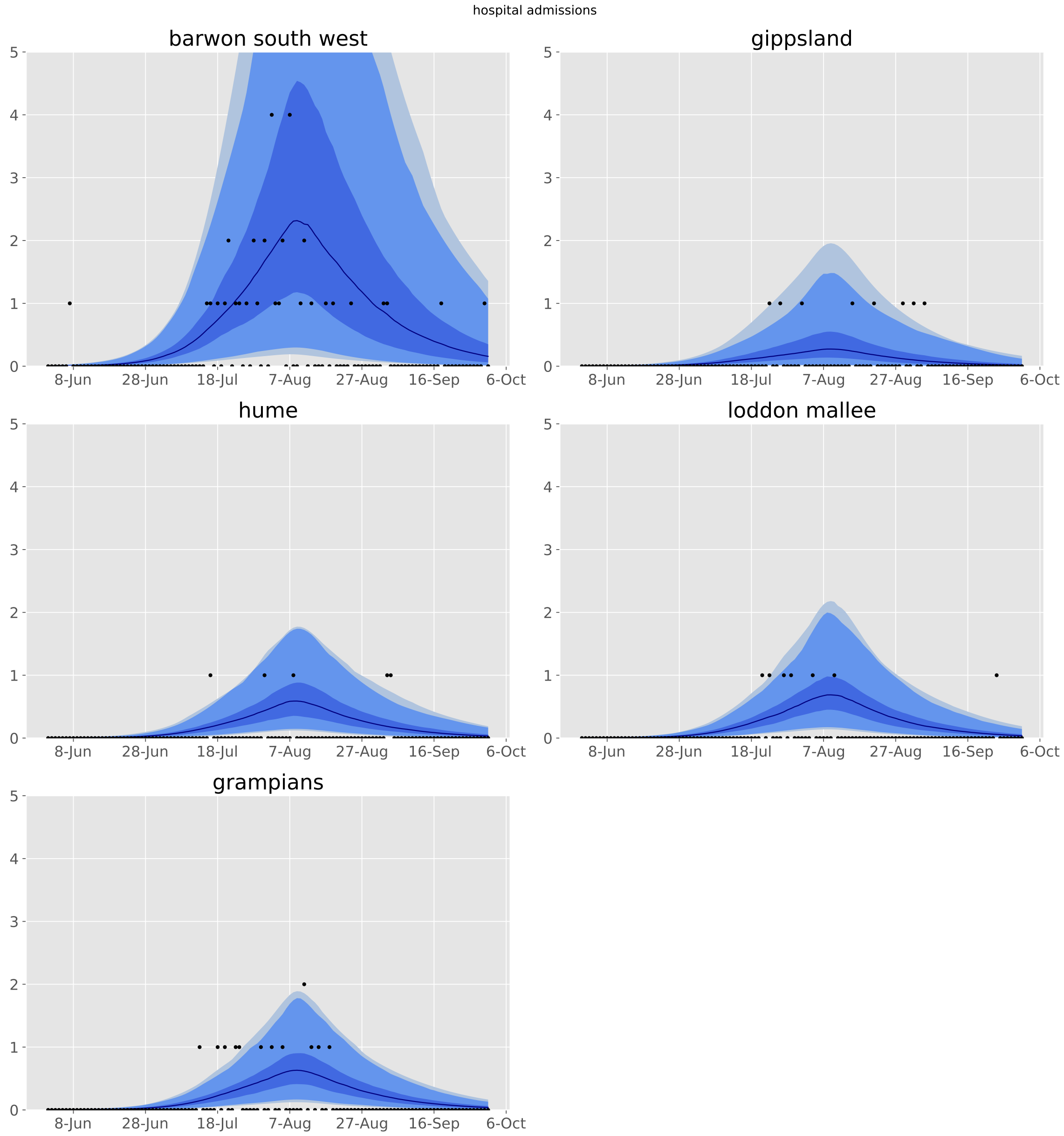

Figure 10: ***Validation fit to daily time series of hospitalisations for each regional health service cluster.*** *Daily hospitalisation (black dots) overlaid on the median modelled hospitalisations (dark blue line), with shaded areas representing the 25th to 75th centile (mid blue), 2.5^th^ to 97.5^th^ centile (light blue) and 1st to 99th centile (faintest blue) of estimated hospitalisations.*

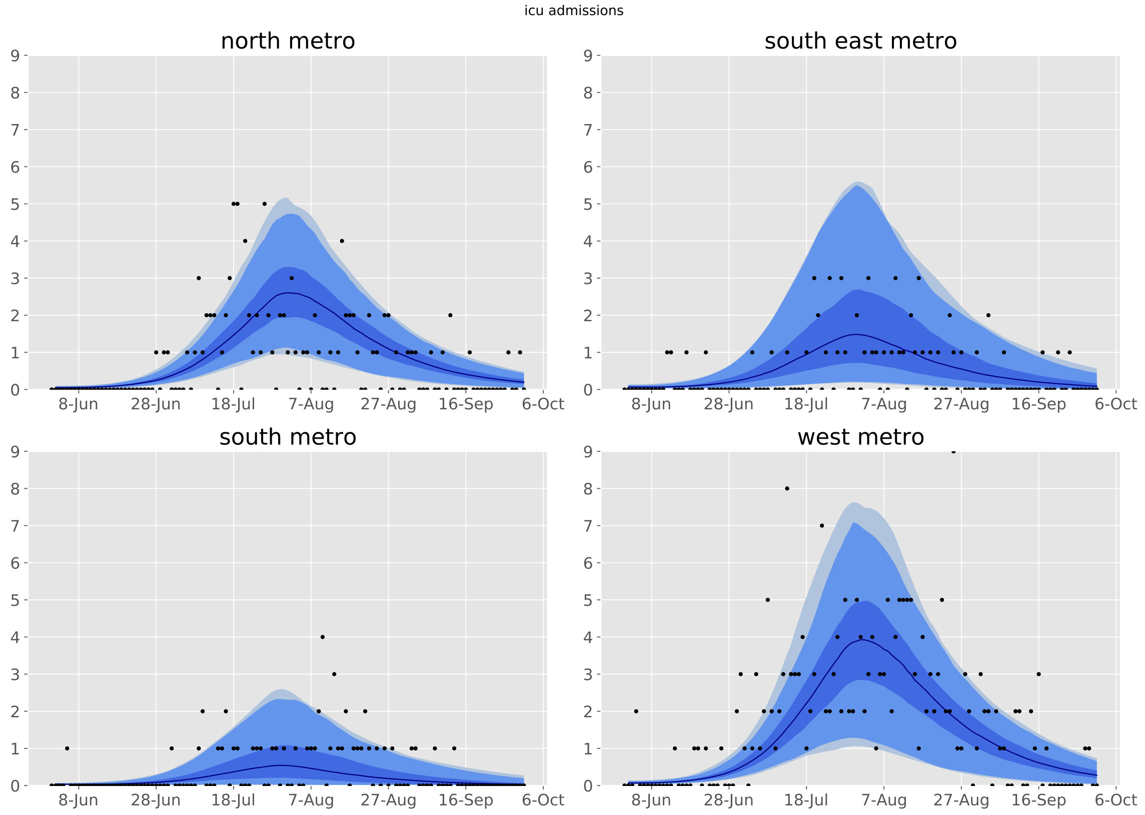

Figure 11: ***Validation fit to ICU occupancy for each metropolitan health service cluster.*** *ICU occupancy (black dots) overlaid on the median modelled ICU occupancy (dark blue line), with shaded areas representing the 25th to 75th centile (mid blue), 2.5th to 97.5th centile (light blue) and 1st to 99th centile (faintest blue) of estimated ICU occupancy.*

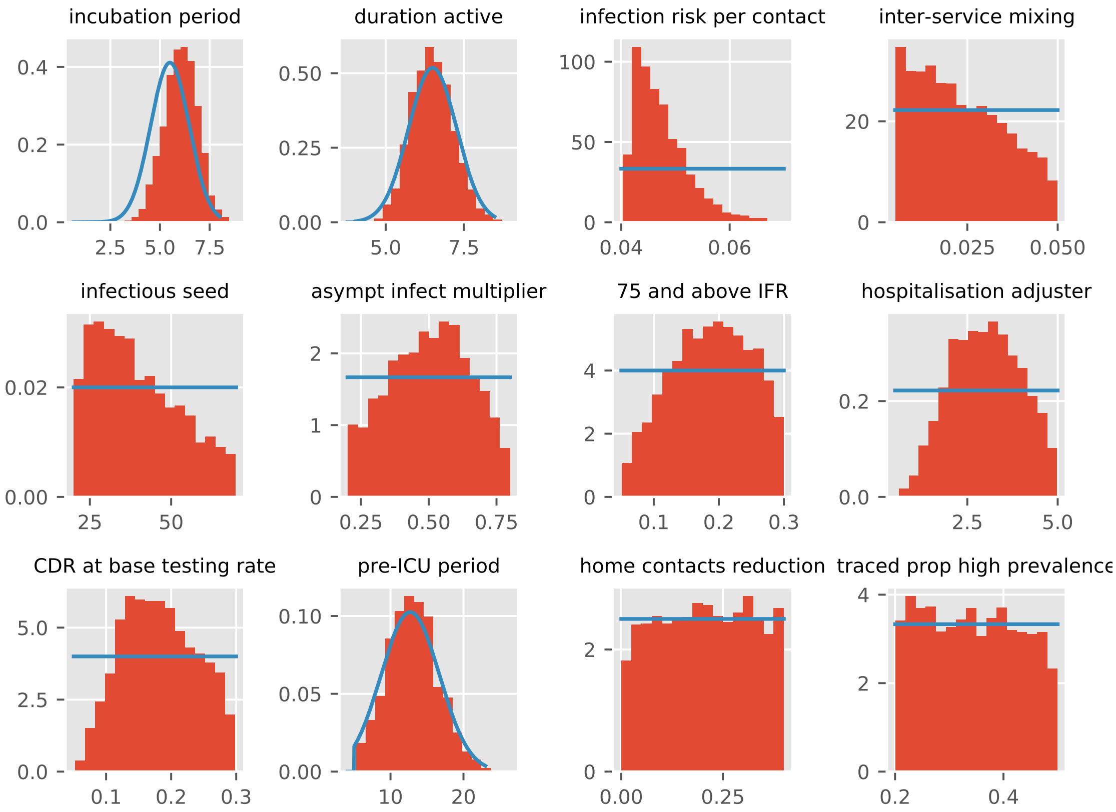

Figure 12: ***Histograms of state-wide epidemiological parameter posteriors, other than key parameters of interest*** *(presented in main manuscript).*

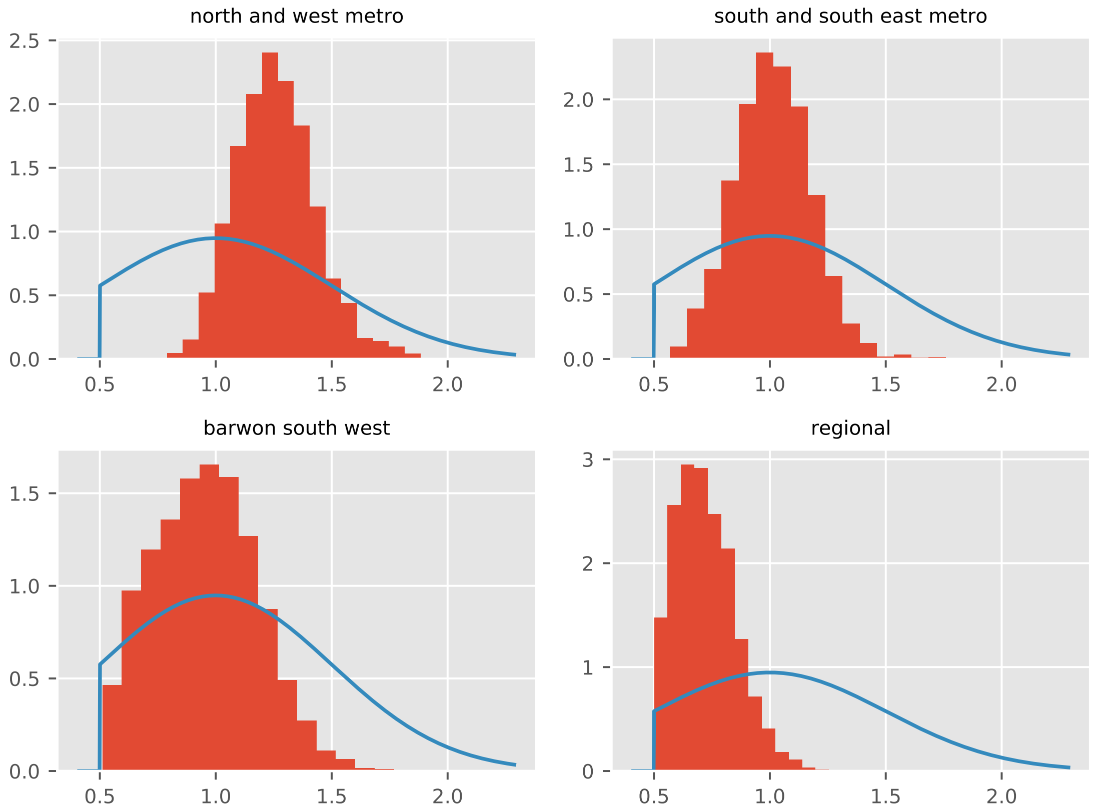

Figure 13: ***Histograms of service-specific contact rate modifier parameter posteriors.***

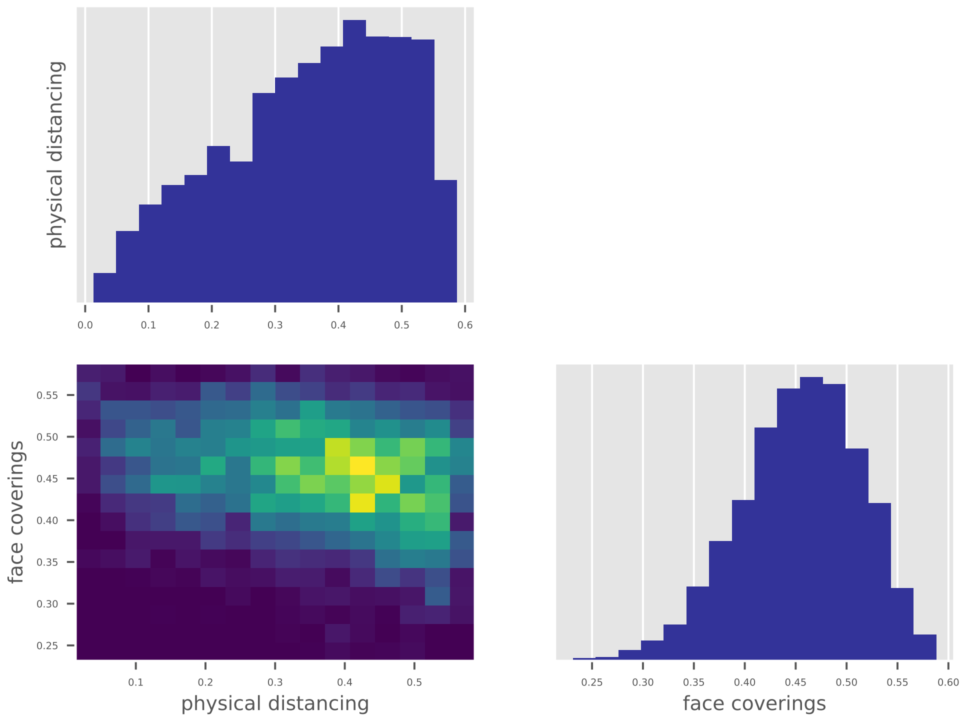

Figure 14: ***Correlation matrix for key epidemiological parameters of interest.***

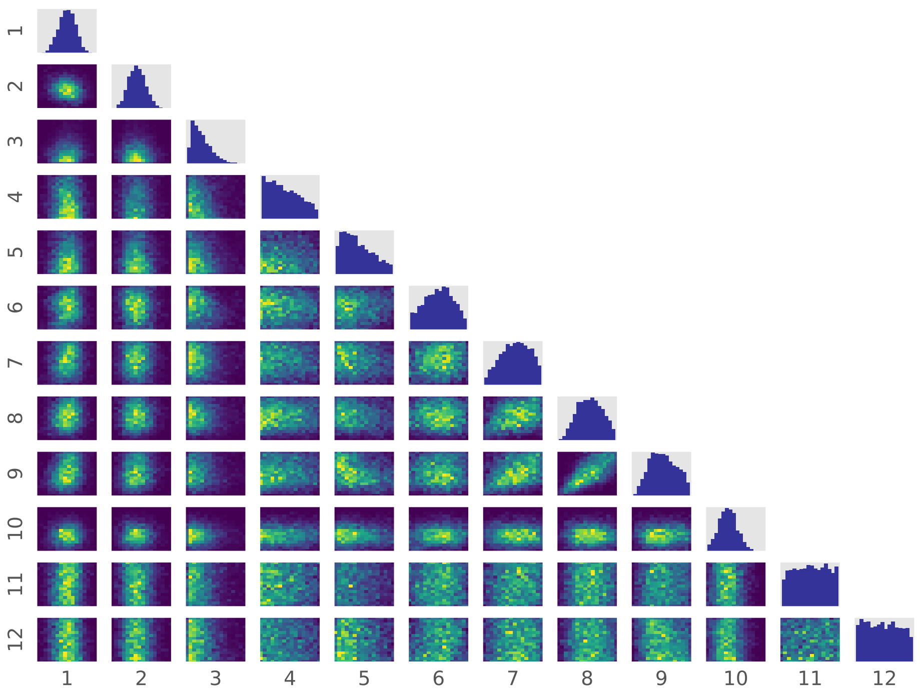

Figure 15: ***Correlation matrix for other state-wide epidemiological parameters.*** *Key: 1, incubation period; 2, duration active; 3, infection risk per contact; 4, inter-service mixing; 5, infectious seed; 6, asympt infect multiplier; 7, 75 and above IFR; 8, hospitalisation adjuster; 9, CDR at base testing rate; 10, pre-ICU period; 11, home contacts reduction; 12, traced prop at high prevalence.*

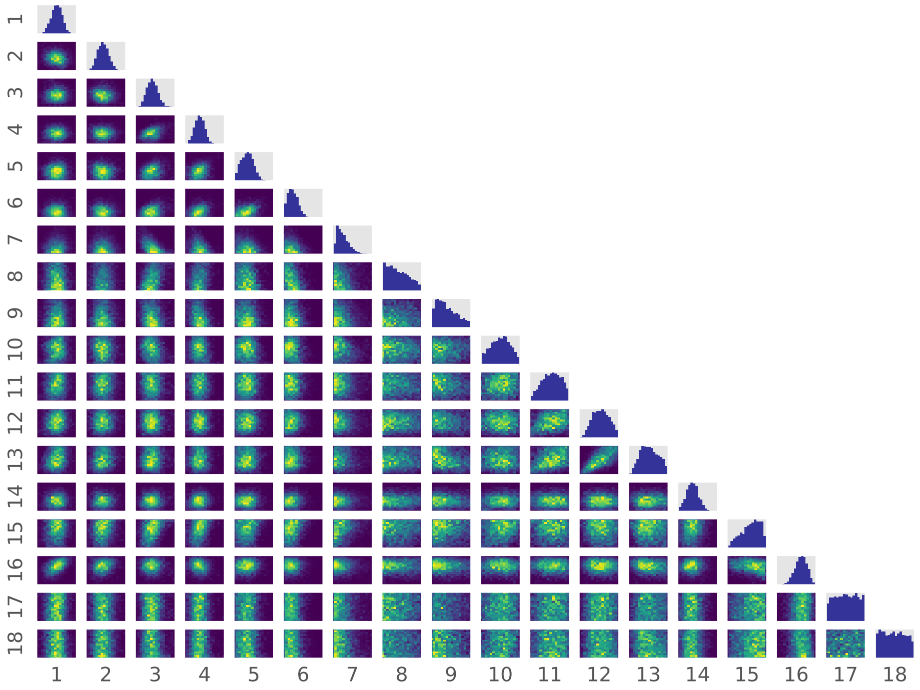

Figure 16: ***Correlation matrix for all parameters.*** *Key: 1, incubation period; 2, duration active; 3, north and west metro; 4, south and south east metro; 5, barwon south west; 6, regional; 7, infection risk per contact; 8, inter-service mixing; 9, infectious seed; 10, asympt infect multiplier; 11, 75 and above IFR; 12, hospitalisation adjuster; 13, CDR at base testing rate; 14, pre-ICU period; 15, physical distancing; 16, face coverings; 17, home contacts reduction; 18, traced prop at high prevalence.*

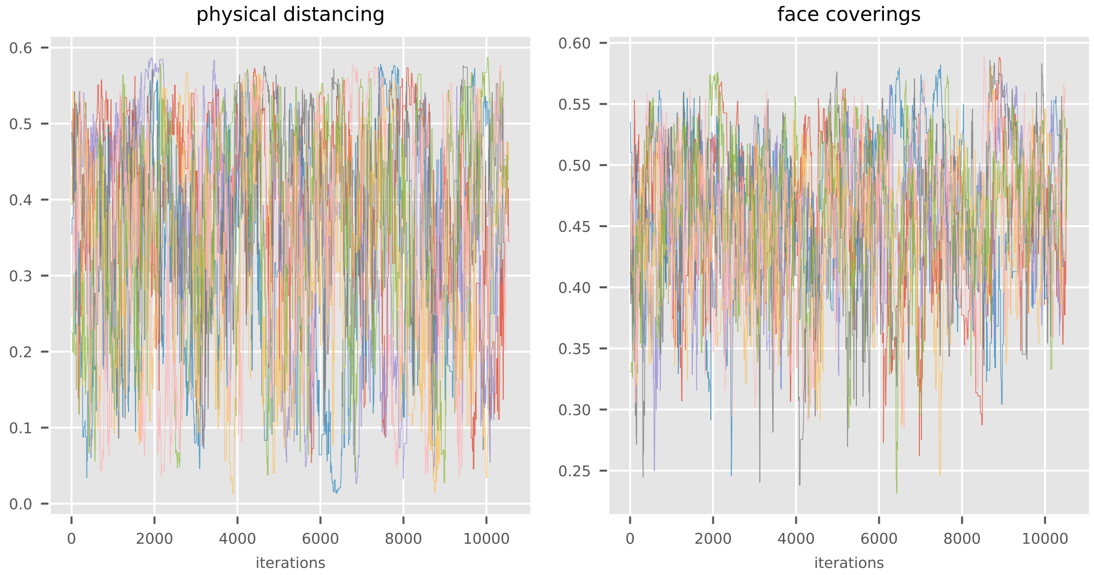

Figure 17: ***Parameter progression traces for key estimation parameters.***

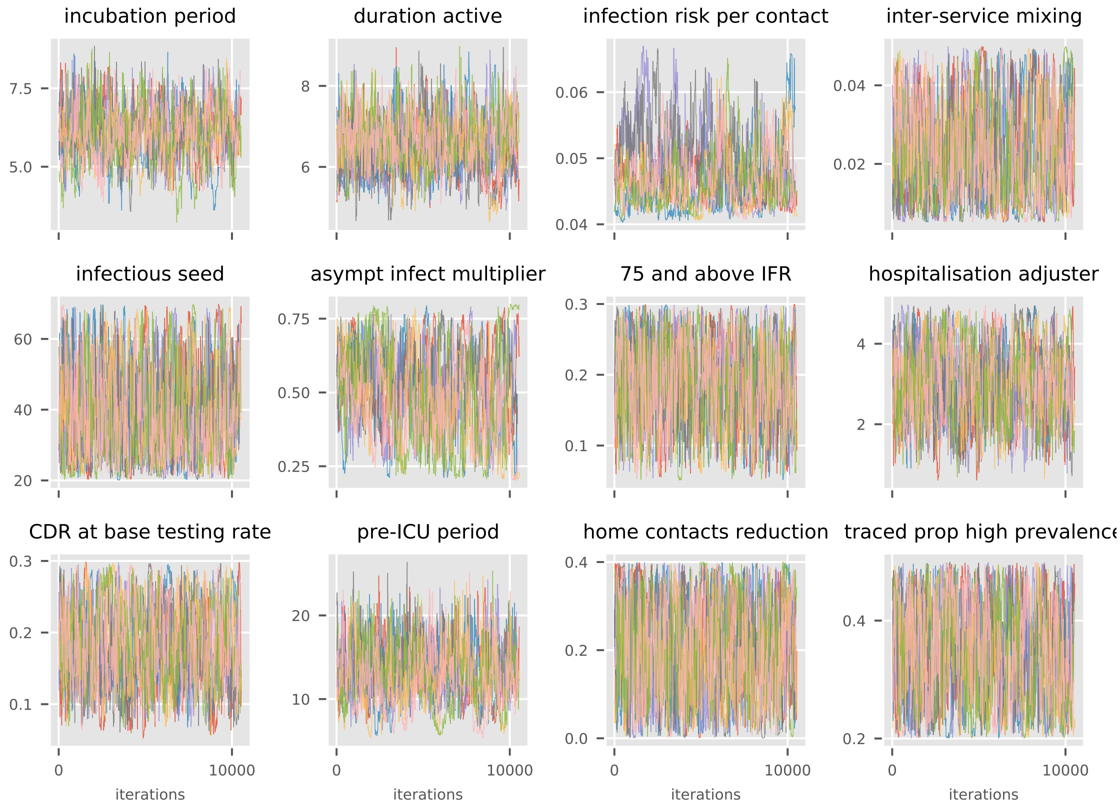

Figure 18: ***Parameter progression traces for epidemiological parameters.***

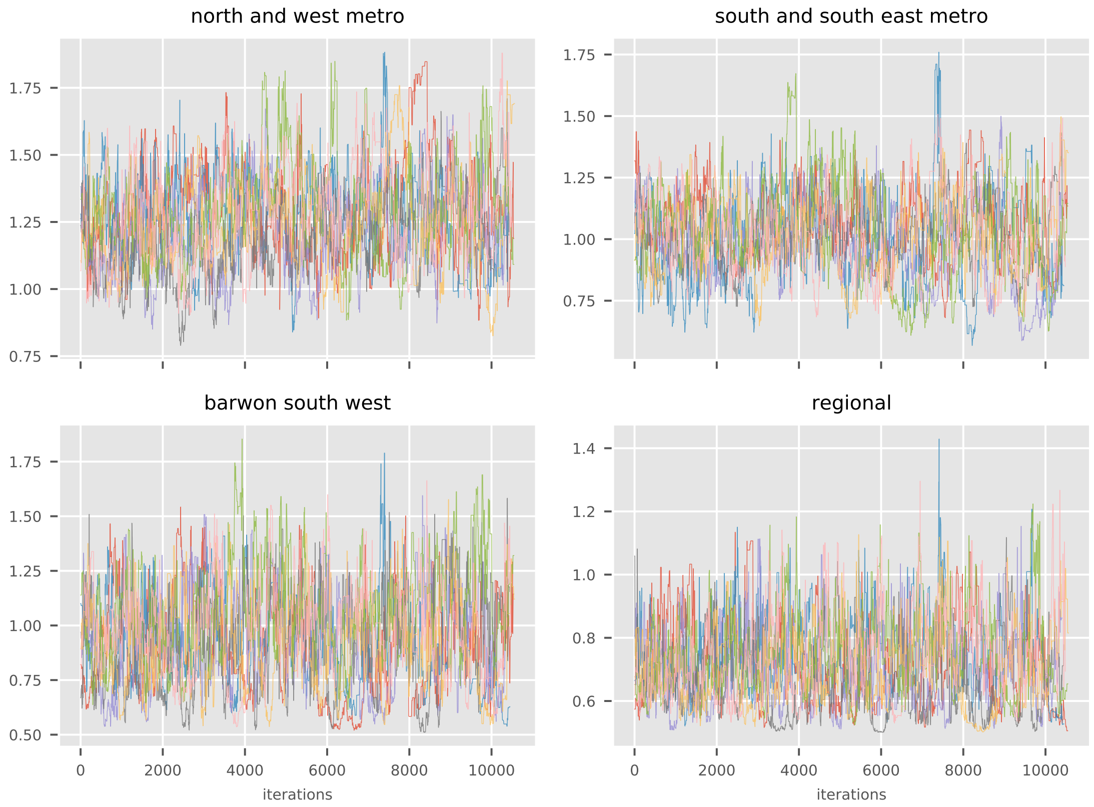

Figure 19: ***Parameter progression traces for service contact rate modifier parameters.***

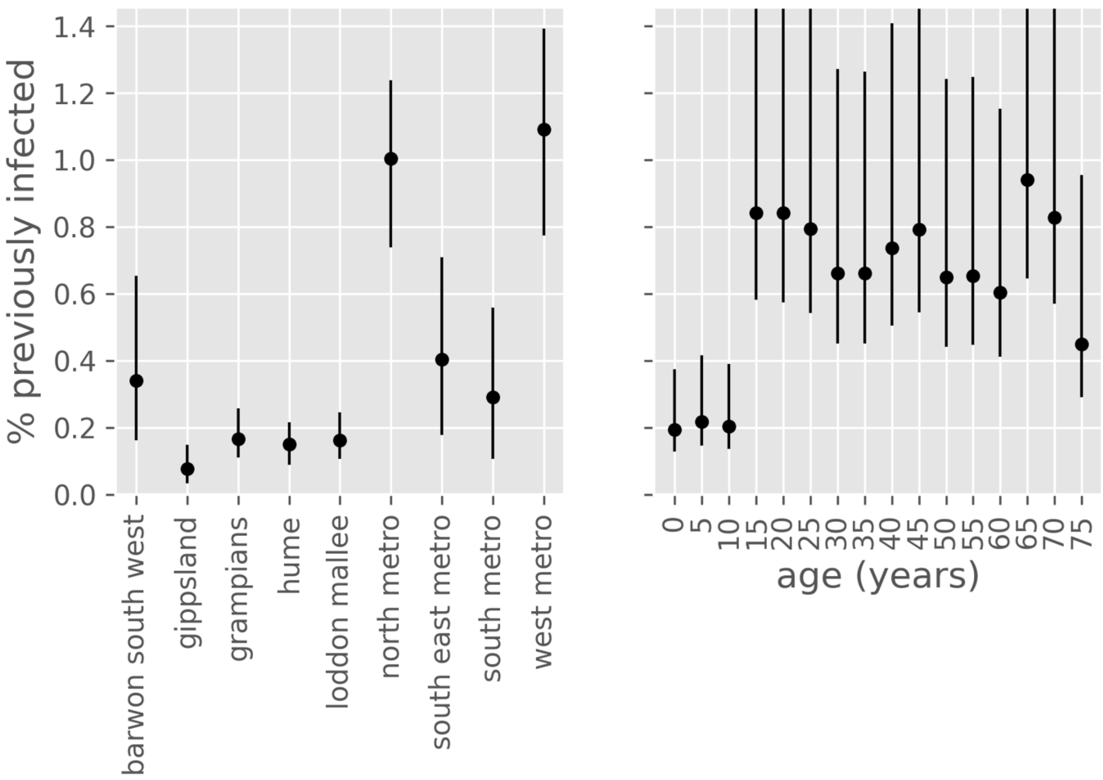

Figure 20: ***Estimated proportion of population recovered from COVID-19 at 1st October 2020, by age group and health service cluster.*** *Point estimates with associated 50% credible intervals. (This quantity is similar to an attack rate, except with deaths excluded from the denominator. Infections from first wave not considered.)*

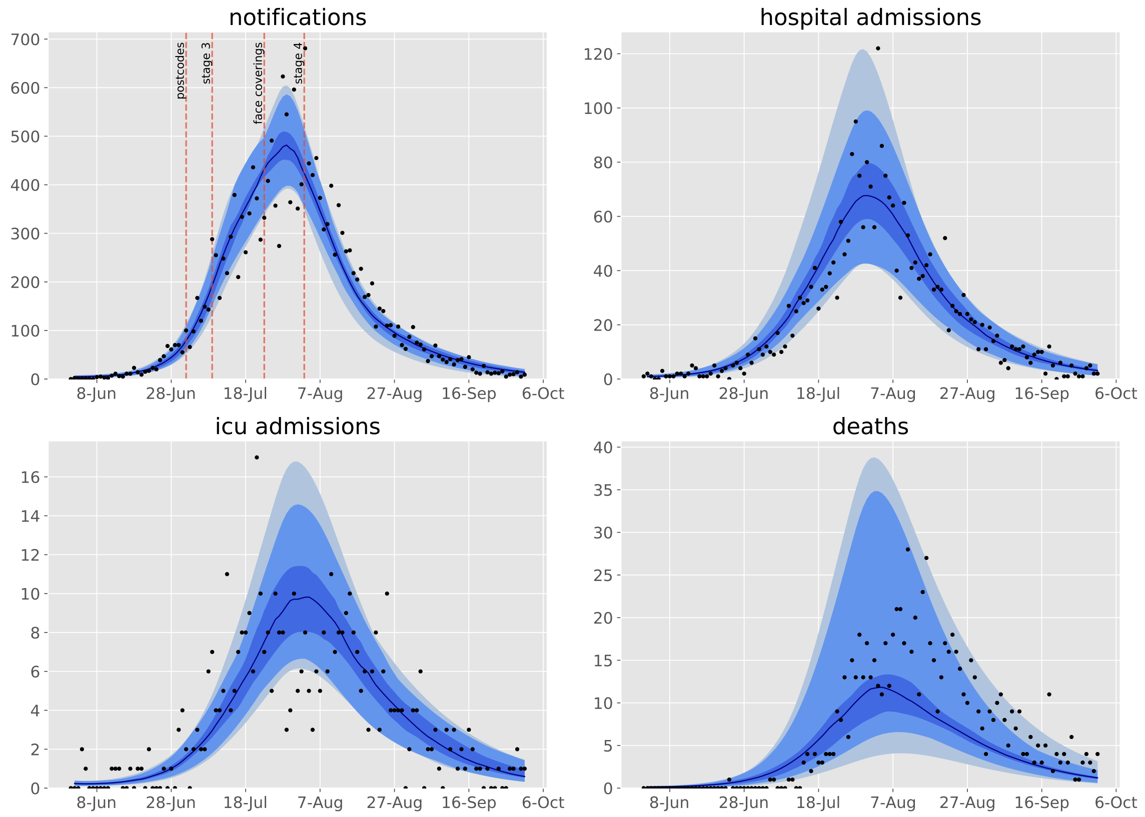

Figure 21: ***Model fit to statewide indicators under sensitivity analysis using matrices derived using data from Belgium.*** *Daily confirmed cases (black dots) overlaid on the median modelled detected cases (dark blue line), with shaded areas representing the 25th to 75th centile (mid blue), 2.5th to 97.5th centile (light blue) and 1st to 99th centile (faintest blue) of estimated detected cases.*

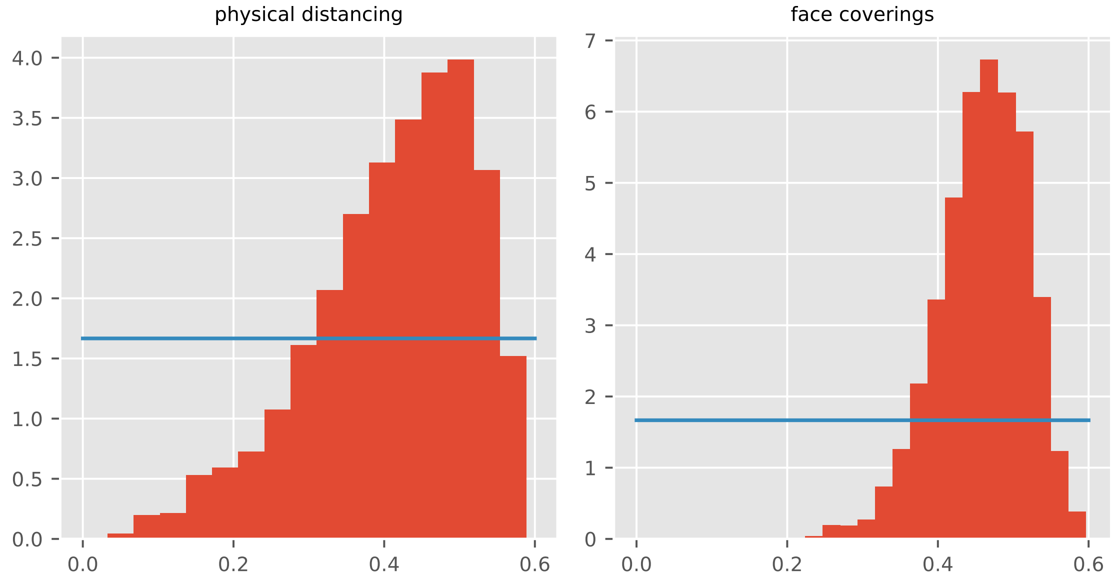

Figure 22: ***Posterior distributions of key model parameters under sensitivity analysis using matrices derived using data from Belgium.***

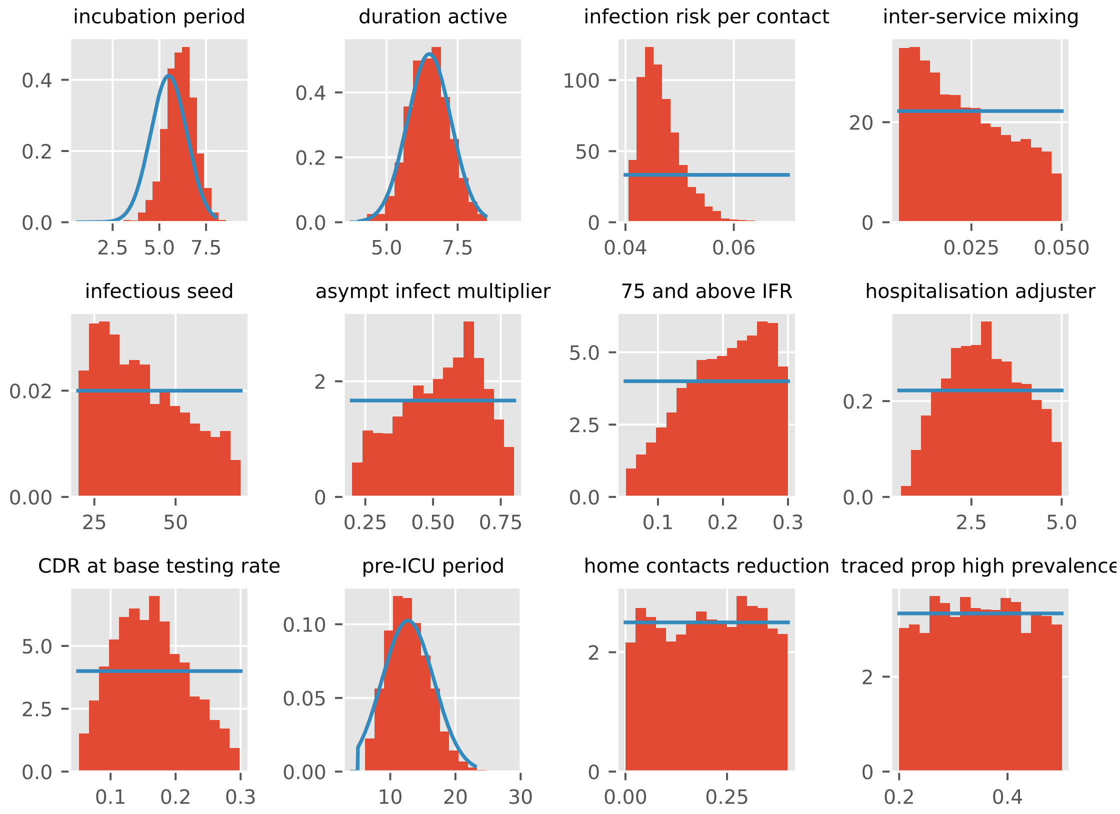

Figure 23: ***Posterior distributions of epidemiological parameters under sensitivity analysis using matrices derived using data from Belgium***

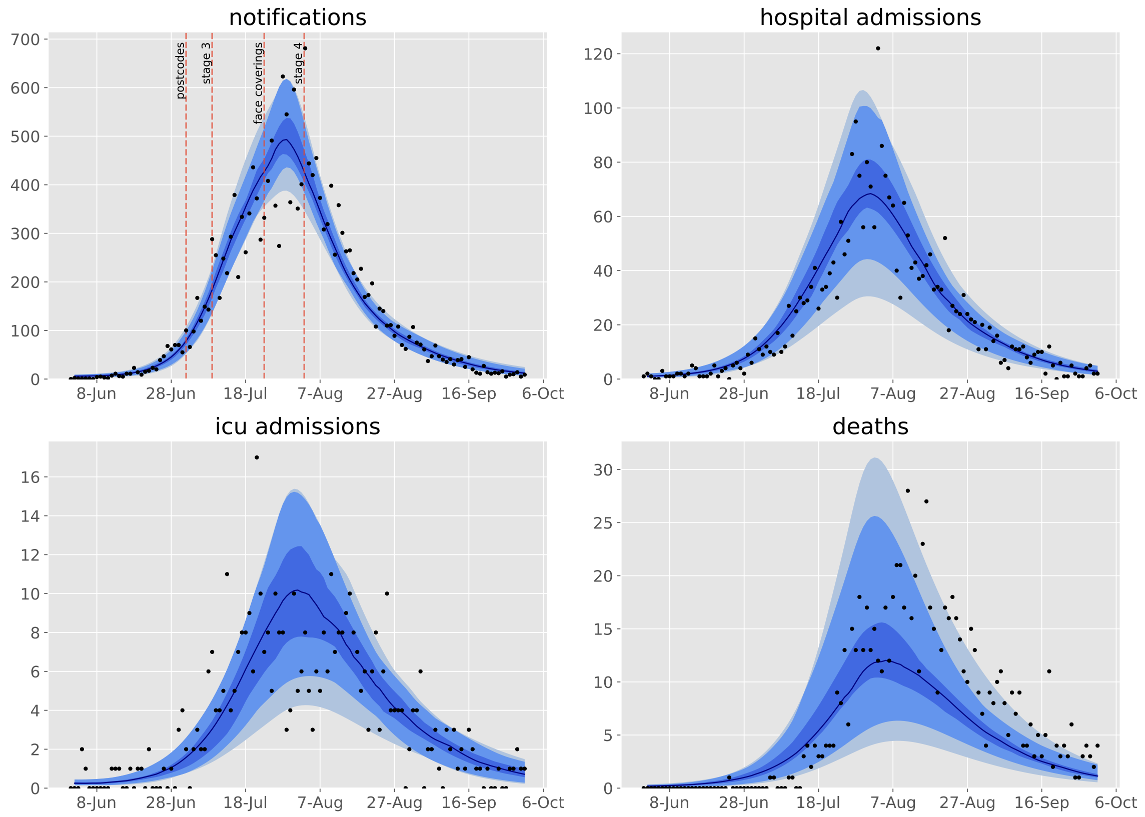

Figure 24: ***Model fit to statewide indicators under sensitivity analysis mapping Google residential mobility to home locations of mixing matrices.*** *Daily confirmed cases (black dots) overlaid on the median modelled detected cases (dark blue line), with shaded areas representing the 25th to 75th centile (mid blue), 2.5th to 97.5th centile (light blue) and 1st to 99th centile (faintest blue) of estimated detected cases.*

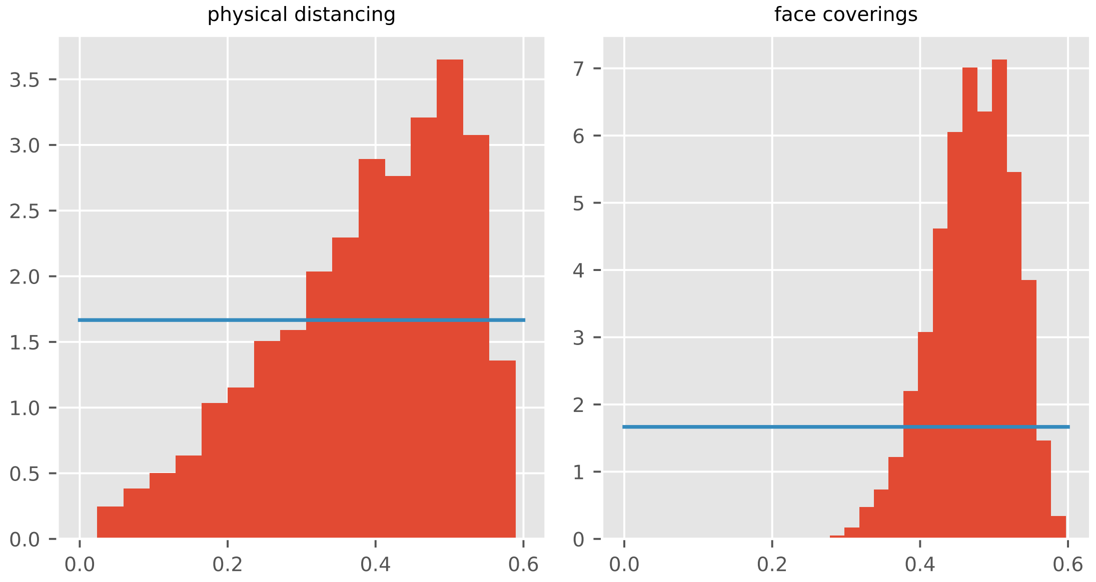

Figure 25: ***Posterior distributions of key model parameters under sensitivity analysis mapping Google residential mobility to home locations of mixing matrices.***

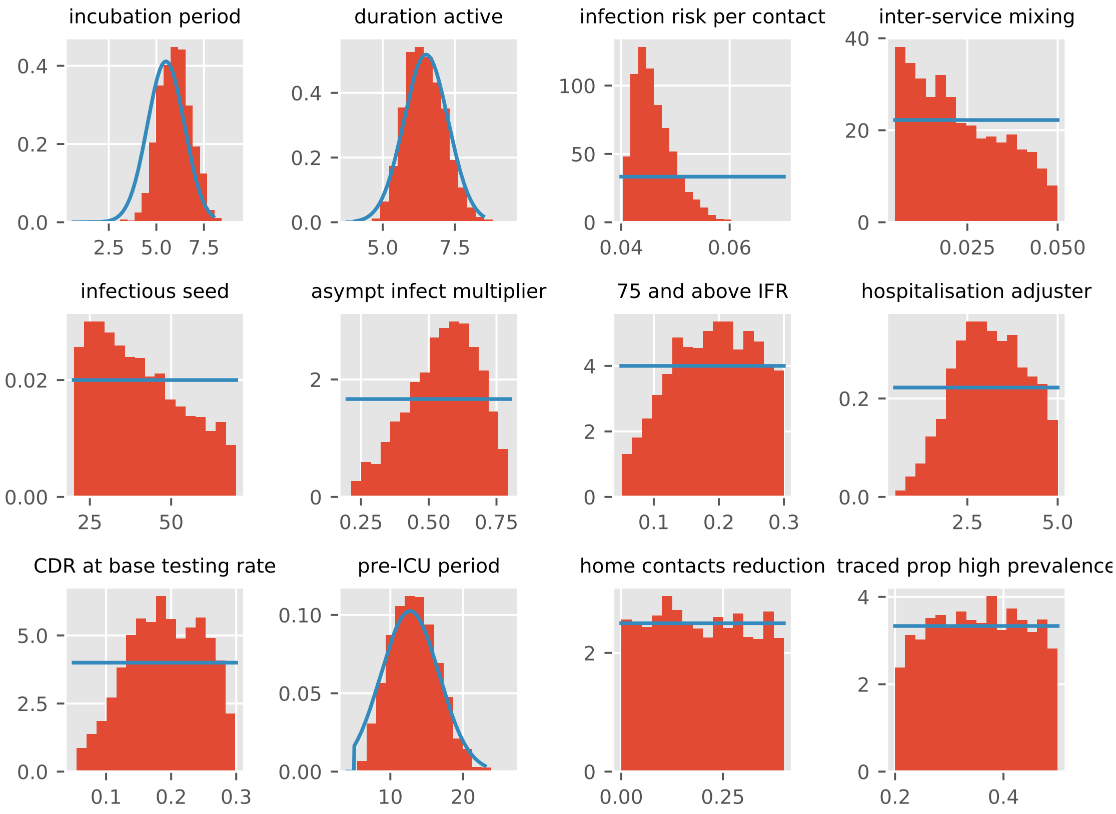

Figure 26: ***Posterior distributions of epidemiological parameters under sensitivity analysis mapping Google residential mobility to home locations of mixing matrices.***

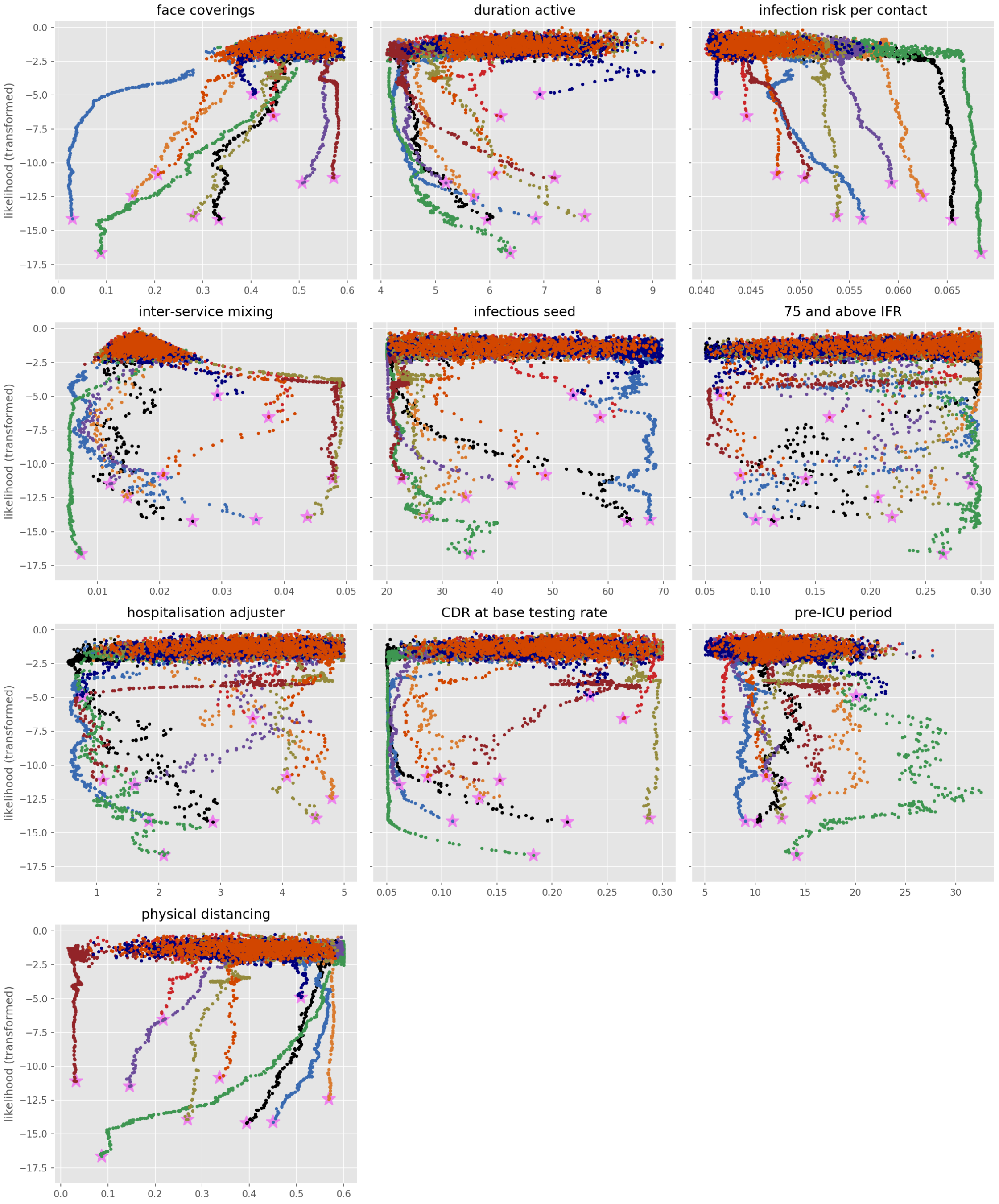

Figure 27: ***Likelihood against ten key parameters during calibration initialised with Latin Hypercube Sampling.*** *The different colours represent the ten independent adaptive Metropolis chains initialised with Latin Hypercube Sampling. The likelihood values were transformed to increase clarity using $x \to -log(-log(x) + m + 1)$, where m is the maximum log-likelihood value. The pink stars highlight the starting points.*

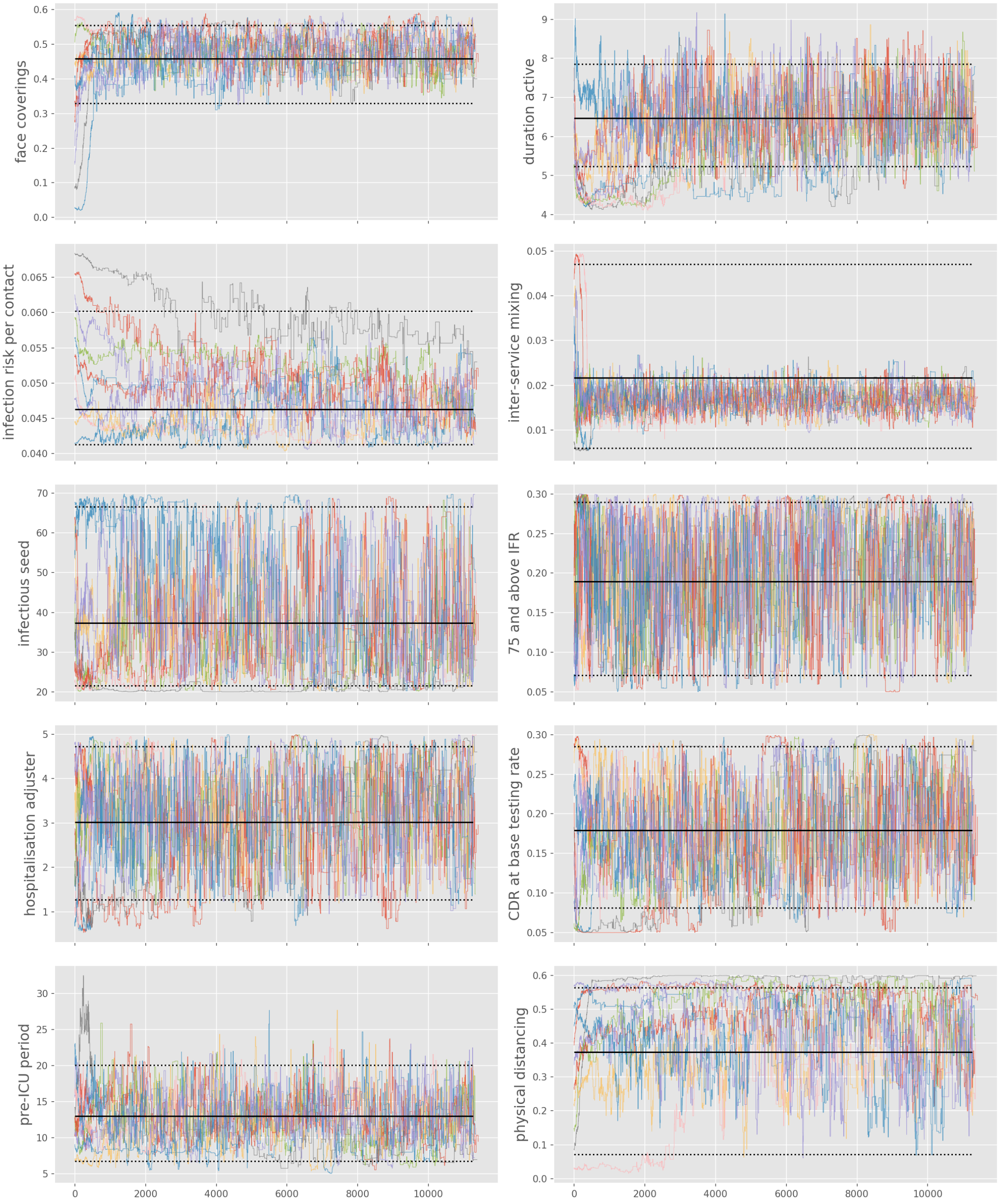

Figure 28: ***Parameter progression traces during calibration initialised with Latin Hypercube Sampling.*** *The horizontal lines represent the median (solid line) and 95% credible interval (dotted lines) of the posterior estimates obtained in the main analysis.*

References

[1] Trauer, J. M., Ragonnet, R., Doan, T. N. & McBryde, E. S. Modular programming for tuberculosis control, the "autumn" platform. *BMC Infect Dis* **17**, 546 (2017). URL https://www.ncbi.nlm.nih.gov/pubmed/28784094.

[2] Caldwell, J. M. *et al.* Understanding covid-19 dynamics and the effects of interventions in the philippines: A mathematical modelling study. *The Lancet Regional Health – Western Pacific* **14** (2021). URL https://doi.org/10.1016/j.lanwpc.2021.100211.

[3] McAloon, C. *et al.* Incubation period of covid-19: a rapid systematic review and meta-analysis of observational research. *BMJ Open* **10**, e039652 (2020). URL https://www.ncbi.nlm.nih.gov/pubmed/32801208.

[4] Mossong, J. *et al.* Social contacts and mixing patterns relevant to the spread of infectious diseases. *PLOS Medicine* **5**, e74 (2008). URL https://doi.org/10.1371/journal.pmed.0050074.

[5] O'Driscoll, M. *et al.* Age-specific mortality and immunity patterns of sars-cov-2. *Nature* **590**, 140–145 (2021). URL https://www.ncbi.nlm.nih.gov/pubmed/33137809.

[6] Zhang, J. *et al.* Changes in contact patterns shape the dynamics of the covid-19 outbreak in china. *Science* **368**, 1481–1486 (2020). URL https://www.ncbi.nlm.nih.gov/pubmed/32350060.

[7] Lauer, S. A. *et al.* The incubation period of coronavirus disease 2019 (covid-19) from publicly reported confirmed cases: Estimation and application. *Ann Intern Med* **172**, 577–582 (2020). URL https://www.ncbi.nlm.nih.gov/pubmed/32150748.

[8] Li, Q. *et al.* Early transmission dynamics in wuhan, china, of novel coronavirus-infected pneumonia. *N Engl J Med* **382**, 1199–1207 (2020). URL https://www.ncbi.nlm.nih.gov/pubmed/31995857.

[9] Bi, Q. *et al.* Epidemiology and transmission of covid-19 in 391 cases and 1286 of their close contacts in shenzhen, china: a retrospective cohort study. *Lancet Infect Dis* **20**, 911–919 (2020). URL https://www.ncbi.nlm.nih.gov/pubmed/32353347.

[10] Meehan, M. T. *et al.* Modelling insights into the covid-19 pandemic. *Paediatr Respir Rev* **35**, 64–69 (2020). URL https://www.ncbi.nlm.nih.gov/pubmed/32680824.

[11] He, X. *et al.* Temporal dynamics in viral shedding and transmissibility of covid-19. *Nat Med* **26**, 672–675 (2020). URL https://www.ncbi.nlm.nih.gov/pubmed/32296168.

[12] Byrne, A. W. *et al.* Inferred duration of infectious period of sars-cov-2: rapid scoping review and analysis of available evidence for asymptomatic and symptomatic covid-19 cases. *BMJ Open* **10**, e039856 (2020). URL https://www.ncbi.nlm.nih.gov/pubmed/32759252.

[13] Pritchard, M. G., Olliaro, P. L. & Group, I. C. C. Symptoms at presentation for patients admitted to hospital with covid-19: results from the isaric prospective multinational observational study. *medRxiv* (2020). URL https://www.ncbi.nlm.nih.gov/pubmed/33140062.

[14] Baillie, J. K. *et al.* Isaric clinical data report 8 april 2021. *medRxiv* 2020.07.17.20155218 (2021). URL https://www.medrxiv.org/content/10.1101/2020.07.17.20155218v8.full.pdf.

[15] Sah, P. *et al.* Asymptomatic sars-cov-2 infection: A systematic review and meta-analysis. *Proceedings of the National Academy of Sciences* **118**, e2109229118 (2021). URL http://www.pnas.org/content/118/34/e2109229118.abstract.

[16] Pollán, M. *et al.* Sars-cov-2 seroprevalence in spain - authors' reply. *Lancet* **396**, 1484–1485 (2020). URL https://www.ncbi.nlm.nih.gov/pubmed/33160561.

[17] (2020). URL https://www.rivm.nl/archief-weekrapportages-covid-19-in-nederland.

[18] Haario, H., Saksman, E. & Tamminen, J. An adaptive metropolis algorithm. *Bernoulli* **7** (2001).
