# Supplementary material for: Understanding how Victoria, Australia gained control of its second COVID-19 wave
Source: Nat Commun. 2021 Nov 1;12:6266. doi: 10.1038/s41467-021-26558-4 (PMC8560916; doi:10.1038/s41467-021-26558-4)
Supplement: Supplementary file 3 — Reporting Summary [file 41467_2021_26558_MOESM3_ESM.pdf]

nature research

Corresponding author(s): James Trauer

Last updated by author(s): Apr 30, 2021

Reporting Summary

Nature Research wishes to improve the reproducibility of the work that we publish. This form provides structure for consistency and transparency in reporting. For further information on Nature Research policies, see our Editorial Policies and the Editorial Policy Checklist.

Statistics

For all statistical analyses, confirm that the following items are present in the figure legend, table legend, main text, or Methods section.

n/a	Confirmed	
☐	☒	The exact sample size (*n*) for each experimental group/condition, given as a discrete number and unit of measurement
☒	☐	A statement on whether measurements were taken from distinct samples or whether the same sample was measured repeatedly
☒	☐	The statistical test(s) used AND whether they are one- or two-sided<br>*Only common tests should be described solely by name; describe more complex techniques in the Methods section.*
☒	☐	A description of all covariates tested
☒	☐	A description of any assumptions or corrections, such as tests of normality and adjustment for multiple comparisons
☐	☒	A full description of the statistical parameters including central tendency (e.g. means) or other basic estimates (e.g. regression coefficient) AND variation (e.g. standard deviation) or associated estimates of uncertainty (e.g. confidence intervals)
☒	☐	For null hypothesis testing, the test statistic (e.g. *F*, *t*, *r*) with confidence intervals, effect sizes, degrees of freedom and *P* value noted<br>*Give P values as exact values whenever suitable.*
☐	☒	For Bayesian analysis, information on the choice of priors and Markov chain Monte Carlo settings
☒	☐	For hierarchical and complex designs, identification of the appropriate level for tests and full reporting of outcomes
☒	☐	Estimates of effect sizes (e.g. Cohen's *d*, Pearson's *r*), indicating how they were calculated

Our web collection on statistics for biologists contains articles on many of the points above.

Software and code

Policy information about availability of computer code

Data collection	The Google mobility input data are available through our GitHub and Zenodo repositories, as indicated below (in the repository folder data/ inputs). Calibration data (disaggregated by health service and date only) are stored in encrypted form.
Data analysis	All model code is available at https://github.com/monash-emu/AuTuMN. The results presented in the manuscript were obtained from the code run from git tag https://github.com/monash-emu/AuTuMN/releases/tag/vic_2nd_revision and are also deposited at https://doi.org/10.5281/zenodo.5553690.<br>Dependencies include a number of standard Python packages available at pypi.org, along with our package for compartmental model construction (available at https://github.com/monash-emu/summer, with documentation available at http://summerepi.com/index.html). The age-specific mixing matrices were created using the socialmixr package for R, version 0.1.8.

For manuscripts utilizing custom algorithms or software that are central to the research but not yet described in published literature, software must be made available to editors and reviewers. We strongly encourage code deposition in a community repository (e.g. GitHub). See the Nature Research guidelines for submitting code & software for further information.

Data

Policy information about availability of data

All manuscripts must include a data availability statement. This statement should provide the following information, where applicable:

- Accession codes, unique identifiers, or web links for publicly available datasets
- A list of figures that have associated raw data
- A description of any restrictions on data availability

All the code and data needed to reproduce the analysis are present on our Git repository, with calibration targets stored in encrypted form. Note that statewide estimates are numerically similar to that available at covid19data.com.au. All output data from the model calibration presented are available at http://

www.autumn-data.com/app/covid_19/region/victoria/run/1627427875-54bf2e3.html. Under the terms of our data sharing agreement with the Victorian Department of Health, all calibration targets are saved in our repository in encrypted form. These values are similar to those publicly available, including at covid19data.com.au

Field-specific reporting

Please select the one below that is the best fit for your research. If you are not sure, read the appropriate sections before making your selection.

- [x] Life sciences
- [ ] Behavioural & social sciences
- [ ] Ecological, evolutionary & environmental sciences

For a reference copy of the document with all sections, see nature.com/documents/nr-reporting-summary-flat.pdf

Life sciences study design

All studies must disclose on these points even when the disclosure is negative.

Sample size	Not applicable to this study design, which considers the entire population of the jurisdiction (Victoria).
Data exclusions	All calibration data available from the simulation period were used as calibration targets, as described in Supplement Section 10, Calibration. No data were excluded.
Replication	The full analysis can be replicated from the code on our repository, running from the appropriate commit ID.
Randomization	Not applicable to this modelling analysis, which did not compare across patient groups.
Blinding	Not applicable to this modelling analysis, which did not assign patients to treatment groups.

Reporting for specific materials, systems and methods

We require information from authors about some types of materials, experimental systems and methods used in many studies. Here, indicate whether each material, system or method listed is relevant to your study. If you are not sure if a list item applies to your research, read the appropriate section before selecting a response.

Materials & experimental systems

n/a	Involved in the study	
x		Antibodies
x		Eukaryotic cell lines
x		Palaeontology and archaeology
x		Animals and other organisms
x		Human research participants
x		Clinical data
x		Dual use research of concern

Methods

n/a	Involved in the study	
x		ChIP-seq
x		Flow cytometry
x		MRI-based neuroimaging
